# Dynamic changes in lung responses after single and repeated exposures to cigarette smoke in mice

**DOI:** 10.1371/journal.pone.0212866

**Published:** 2019-02-28

**Authors:** Michelle L. Engle, Justine N. Monk, Corey M. Jania, Jessica R. Martin, John C. Gomez, Hong Dang, Joel S. Parker, Claire M. Doerschuk

**Affiliations:** 1 Marsico Lung Institute, University of North Carolina, Chapel Hill, NC, United States of America; 2 Curriculum in Genetics and Molecular Biology, University of North Carolina, Chapel Hill, NC, United States of America; 3 Pathobiology and Translational Science Graduate Program, University of North Carolina, Chapel Hill, NC, United States of America; 4 Division of Pulmonary Diseases and Critical Care Medicine, University of North Carolina, Chapel Hill, NC, United States of America; 5 Department of Medicine, University of North Carolina, Chapel Hill, NC, United States of America; 6 Department of Genetics, University of North Carolina, Chapel Hill, NC, United States of America; 7 Lineberger Comprehensive Cancer Center, University of North Carolina, Chapel Hill, NC, United States of America; Institute of Lung Biology and Disease (iLBD), Helmholtz Zentrum München, GERMANY

## Abstract

Cigarette smoke is well recognized to cause injury to the airways and the alveolar walls over time. This injury usually requires many years of exposure, suggesting that the lungs may rapidly develop responses that initially protect it from this repetitive injury. Our studies tested the hypotheses that smoke induces an inflammatory response and changes in mRNA profiles that are dependent on sex and the health status of the lung, and that the response of the lungs to smoke differs after 1 day compared to 5 days of exposure. Male and female wildtype (WT) and *Scnn1b*-transgenic (βENaC) mice, which have chronic bronchitis and emphysematous changes due to dehydrated mucus, were exposed to cigarette smoke or sham air conditions for 1 or 5 days. The inflammatory response and gene expression profiles were analyzed in lung tissue. Overall, the inflammatory response to cigarette smoke was mild, and changes in mediators were more numerous after 1 than 5 days. βENaC mice had more airspace leukocytes than WT mice, and smoke exposure resulted in additional significant alterations. Many genes and gene sets responded similarly at 1 and 5 days: genes involved in oxidative stress responses were upregulated while immune response genes were downregulated. However, certain genes and biological processes were regulated differently after 1 compared to 5 days. Extracellular matrix biology genes and gene sets were upregulated after 1 day but downregulated by 5 days of smoke compared to sham exposure. There was no difference in the transcriptional response to smoke between WT and βENaC mice or between male and female mice at either 1 or 5 days. Taken together, these studies suggest that the lungs rapidly alter gene expression after only one exposure to cigarette smoke, with few additional changes after four additional days of repeated exposure. These changes may contribute to preventing lung damage.

## Introduction

Cigarette smoke is a leading health hazard and causes an enormous impact on lung health. Cigarette smoking has long been known to have a significant impact on respiratory health and diseases [1, 2]. Smoking is the number one cause of lung cancer and chronic obstructive pulmonary disease (COPD), and it increases the odds of developing either chronic bronchitis or emphysema [2, 3]. More than 16 million Americans are currently living with a tobacco smoke-related disease, resulting in nearly $170 billion in direct healthcare costs annually [2, 4].

The response of the lungs to the first exposure of cigarette smoke and how this response changes following subsequent exposures is important for understanding tobacco-induced lung injury and is nearly impossible to study in humans. Compared to a single stimulus, the lung’s response to repeated exposures of a stimulus such as endotoxin shows evidence of adaptation or tolerance [5], particularly when the stimulus induces oxidative stress in epithelial and immune cells [6]. The effect of a single exposure to cigarette smoke on gene expression in the lungs has not been evaluated. Most interesting are questions about changes that occur in response to a single dose of cigarette smoke compared to changes resulting from consecutive repeated exposures. The changes in gene expression, and particularly in pathways regulating host defense, can be used to evaluate how the lung adapts to cigarette smoke exposure. Additionally, many mouse models of smoking use acute exposure durations of fewer than 5 consecutive days of cigarette smoke exposure [7, 8]. Understanding how the lungs cope with the oxidant burden and the many gaseous and particulate components of cigarette smoke initially and upon repeated exposures is likely to provide information about pathways and processes underlying host defense and the development of chronic lung disease.

Males and females differ in their response to smoke exposure and the development of tobacco smoke-associated disease, such as COPD [2, 3]. COPD-associated morbidity and mortality are increased among American women compared to men [9, 10]. In fact, the highest prevalence of women with COPD occurs in North America [11]. Women and men with the same COPD burden respond differently; women experiencing more pronounced symptoms and reporting poorer quality of life than their male counterparts [12, 13]. This is true across the life course, and is particularly pronounced in younger women [14]. Women are more likely to develop severe, early onset COPD [15, 16] and are more likely to experience more severe dyspnea than men, despite similar lung function and with fewer pack-years of smoking history [17, 18]. Although the rise in the number of female smokers may contribute to the surge in female COPD prevalence, the difference in lung development and thoracic volume between the sexes may have a role [16]. Importantly, the airway response to smoke is different between males and females [19]. Recently, certain sex-specific genetic risk factors for COPD have been identified for women [20, 21]. These differences between sexes have been shown in humans who are chronic smokers, but no studies investigate sex differences at early time points after initiation of smoking in humans. Additionally, to the best of our knowledge, no publication has assessed the effects of sex on the response of the lungs to acute smoke exposure in a mouse model. Given the differences in smoking-related lung disease in humans, this is an important research question to pursue [20, 22–27]. Understanding how sex impacts the development and initiation of tobacco smoke-related disease is important for developing treatment protocols for patients.

The first exposure of cigarette smoke in humans will not always be to healthy lungs but rather to already inflamed lungs. Airway inflammation is common and has many etiologies. Viruses, other pathogens, environmental factors, and e-cigarettes or other “gateway” tobacco products can each cause airway inflammation. Understanding the impact of airway inflammation on the molecular changes in response to cigarette smoke is critical for our understanding of the effects of smoke in damaged lungs [28, 29]. Our approach to asking questions about the effect of smoke in lungs with inflammation uses transgenic mice overexpressing *Scnn1b*, the gene that codes for the epithelial Na^+^ channel β subunit (βENaC), in the epithelial cells of the airways. βENaC mice have dehydrated airway mucus that results in chronic bronchitis, including mucus cell metaplasia, mucus hypersecretion, mild neutrophilic inflammation, large foamy macrophages, and increased numbers of lymphocytes in both the lumen and the walls of the airways [30–35]. Exposure of neonatal βENaC mice to cigarette smoke enhances airway neutrophilia and mucus production and plugging [36]. In addition to chronic bronchitis, βENaC mice develop an emphysematous phenotype soon after birth. Their distal airspaces become enlarged secondary to obstruction from the pathologically thickened mucus [30]. This development of emphysema in βENaC mice requires upregulation of the metalloproteinase, MMP12, which can degrade alveolar walls [37]. The effects of 1- and 5-day cigarette smoke exposure in the healthy lungs of wild type (WT) mice and the chronically inflamed lungs of βENaC mice were therefore compared.

These studies tested the hypotheses that smoke induces inflammation and changes in mRNA profiles that are dependent on sex (male vs female) and the health status of the lung (chronic bronchitis vs healthy airways), and that the effects of smoke are different after 1 day compared to 5 days of exposure. The inflammatory cells and mediators and the gene expression profiles were measured in the bronchoalveolar lavage (BAL) and lung tissue of male and female WT and βENaC mice after 1- or 5-day exposure to cigarette smoke or air (sham). The changes over time during increasing acute exposure durations are likely to provide insight into the mechanisms important in protection against smoke-induced lung damage.

## Methods

### Mice

Mice were originally obtained from Jackson Laboratories. *Scnn1b*-tg mice were generated and backcrossed to a C57BL/6J background [30, 31]. The colony is maintained by breeding *Scnn1b*-tg mice to WT littermates. These mice were kindly made available to us by Dr. Wanda K. O’Neal. The mice utilized in this study were male and female C57BL/6J WT and *Scnn1b*-tg littermates (βENaC mice) [30]. They were bred and maintained in microisolator cages within ventilated racks in a pathogen-free facility with a 12-hour light/dark cycle and regulated temperature and humidity. Chow and water were provided *ad libitum*. Offspring were genotyped, and WT and βENaC mice were identified. All animal studies were performed in compliance with the U.S. Department of Health and Human Services Guide for the Care and Use of Laboratory Animals. Euthanasia was performed by exposing mice anesthetized with tribromoethanol to a lethal dose of inhaled isoflurane. All studies were reviewed and approved by the Institutional Animal Care and Use Committee at the University of North Carolina at Chapel Hill.

### Smoke exposure system and protocol

Sex-matched 5-7-week old WT and βENaC littermates were exposed to cigarette smoke or sham (room air) exposure. Each exposure and genotype group included both males and females, as described for each study. Exposure occurred in a plexiglass chamber attached to a smoke delivery device using an exposure chamber and smoking machine (inExpose Exposure System, SCIREQ, Chandler, AZ). The chamber contained pie-slice separators and positions for 16 mice. Mice were exposed to mainstream + side-stream smoke from 6 reference cigarettes with filters removed per day (College of Agriculture Reference Cigarette Program, University of Kentucky, 3R4F research cigarettes), a commonly used protocol to allow comparison with other literature [38]. Each cigarette was puffed using the standard Federal Trade Commission smoking machine protocol [38]. The mice received the equivalent of one puff per minute. The sham-exposed control mice were exposed to room air in the exposure chamber for a time equivalent to that needed for active smoke exposure. Mice were exposed to cigarette or sham smoke for 1 day or 5 consecutive days. For studies investigating the inflammatory response, mice were euthanized 16 hours following the end of the 1-day exposure, and 24 hours following the 5th day of exposure in the 5-day exposure groups. For studies of gene profiling, mice were euthanized 4 hours after the completion of the final smoke exposure to assess gene transcription leading to the observed inflammatory response. The right lung was used for microarray gene expression analysis.

### Analysis of inflammatory cells and mediators in BAL

After tying off, removing, and freezing the left lung for PCR analyses of gene expression, the right lung was lavaged three times with cold Dulbecco’s phosphate buffered saline (D-PBS) at a volume adjusted for each mouse’s body weight (microliters D-PBS equal to 0.17% of body weight, in grams). The pooled BAL fluid from each mouse was centrifuged, the supernatant was removed, aliquoted and frozen, and the cells were resuspended in 100uL D-PBS. BAL cells were counted manually with a hemocytometer, and 100,000 cells were transferred onto a cytospin prep and stained (Protocol Hema 3 Stain Set, Fisher Diagnostics) for manual differential counting using light microscopy. Measurement of chemokines, cytokines and other inflammatory mediators were performed using a multiplex ELISA (Bio-Plex Pro Mouse Cytokine 23-Plex Immunoassay), which quantified concentrations of Eotaxin, G-CSF, GM-CSF, IFN-γ, IL-1α, IL-1β, IL-2, IL-3, IL-4, IL-5, IL-6, IL-9, IL-10, IL-12 (p40), IL-12 (p70), IL-13, IL-17A, KC, MCP-1, MIP-1α, MIP-1β, RANTES, and TNFα.

### Preparation of single cell suspensions of lung tissue and flow cytometry

Following BAL collection, a single cell digest was generated from the right lung of each mouse using enzymatic digestion and mechanical disruption, as previously described [39–41]. After lysis to remove red blood cells, the single cell digest was stained to identify neutrophils using CD45 and Ly6G. Stained cells were evaluated using a Beckman Coulter CyAn ADP flow cytometer and analyzed using Summit 4.3 software.

### Gene expression analysis by qPCR

The left lung of each mouse was removed and flash-frozen for RNA isolation. Whole lung tissue was homogenized for isolation of total RNA using a QIAGEN miRNeasy kit. Total RNA was used to make cDNA by reverse transcription polymerase chain reaction. Gene expression was measured using qPCR.

### Gene expression profiling and RNA isolation

In separate studies, lung tissue was obtained 4 hours after 1-day or 5-day sham or smoke exposure and flash frozen (n = 5 animals of each sex per group for all groups with the following exceptions: 1) n = 6 WT and n = 4 βENaC sham 5-day female mice; 2) n = 4 WT smoke-exposed 5-day male mice). RNA was isolated from lung tissue homogenates using the miRNeasy kit (Qiagen). Spectrophotometric ratios of A260/A280 and A260/A230 were 1.7–2.1 and greater than 1.6, respectively. RIN values were greater than 7.4; the average RIN for all samples was 9.1. Affymetrix moGene2.1 array was used for gene expression analysis. Data were evaluated using Affymetrix Expression Console v1.4 software for quality control based on summary statistics, and Partek Genomics Suite v6.6 for normalization. The manufacturer’s quality control thresholds were used (https://tools.thermofisher.com/content/sfs/brochures/exon_gene_arrays_qa_whitepaper.pdf). All samples passed all quality control metrics and were preprocessed and normalized. One sample was identified as mislabeled for smoke exposure based on cotinine concentration in the plasma and gene expression and was removed before proceeding for further analysis. Expression signals from CEL files were preprocessed and normalized by RMA (Robust Multiarray Average) background correction, GC content and probe sequence correction, quantile normalization, and median polish summarization of probe signals mapped to specific genes. Custom probeset-to-gene mappings were generated from Affymetrix Probeset and Transcript Annotation release 35 by consolidating all probesets mapped, in order of preference, to Ensembl 81 gene ID, Refseq mRNA, and Genbank accession numbers. The RMA-normalized log2 intensity values were used as input for analysis with the General Linear Model (glm) function in R.

### Cotinine concentrations in the plasma of mice in which gene expression was studied

Blood was collected from the inferior vena cava using EDTA as the anti-coagulant and centrifuged. Plasma was frozen and stored. Cotinine, a metabolite of nicotine, was measured using an ELISA (Mouse/rat Cotinine ELISA, Calbiotech, Spring Valley, CA, USA), following the manufacturer’s instructions.

### General linear model

The relationship between the gene expression and the variables of interest in the experiment (exposure, genotype, and sex) was modeled using a linear model. The linear model parses out the amount of expression change that is associated with each independent variable, and produces a β coefficient and a p value for each gene and each variable. Each gene may be influenced by one or more of these independent variables at one time–for example, a gene can respond to smoke exposure and also be expressed at different levels in males and females. Thus, a multivariable additive linear model containing the independent variables of smoke vs sham exposures (“exposure”), WT vs βENaC (“genotype”), and male vs female (“sex”) was fit to the gene expression data as the response variable This was performed separately for each exposure duration. The glm method in R was used to fit the expression of each gene (modeled as a normal distribution) and estimate the effect size (β coefficient) of each factor. The β coefficient represents the amount of expression change due to that variable alone. The p values corresponding to the β coefficients, which represent the significance of that effect, were transformed to q values using the Benjamini-Hochberg false discovery method [42] and a false discovery rate (FDR) of 5% was applied to identify significant effects. Genes below the threshold of q<0.05 and with a β coefficient larger than +/-0.379 (fold change >+/-1.3) were designated as “significant”. All code used for linear model analyses is included in S1 File. The full list of all genes and their β coefficients, p values, and q values for each variable at 1 day is included in S2 Table, and the results for 5 days are included in S3 Table.

### Identification of exposure-response genes

Lists of all genes with a significant (q<0.05) effect of exposure and a β coefficient larger than +/-0.379 (fold change >+/-1.3) were compiled separately for 1-day and 5-day exposures. These are referred to as “exposure-response genes”. Genes with a positive β coefficient are upregulated in the smoke-exposed mice, while genes with a negative β coefficient are downregulated in the smoke-exposed mice.

### Overlap analysis

Overlap analysis was used to test for enrichment of GSEA Canonical Pathways processes in gene lists. Custom gene lists derived from each cluster in the heatmap as well as the list of exposure duration-dependent genes were tested for significant overlap with the Canonical Pathways gene sets’ gene lists using the MSigDB overlap computation tool (http://software.broadinstitute.org/gsea/msigdb/help_annotations.jsp#overlap). The hyper-geometric distribution was used to produce statistical estimates of the significance of the overlap.

A custom gene list of the genes different between WT and BENaC, derived from the published results in Saini et al. [35], was tested for overlap with the 1-day and 5-day genotype-response gene lists derived from our analysis. The testGeneOverlap function from the GSA and GeneOverlap libraries, which performs a Fisher’s exact test based on the gene lists input, was used to calculate significance of the overlap.

### Clustering and heatmap generation

K-means clustering was used to group the genes into clusters with distinct expression patterns which were linked to biological functions through evaluation of gene annotations. Hierarchical clustering, using Pearson correlation, was used to group the samples in order to understand the similarities among samples. To determine the number of gene clusters that best describes the dataset, the within-group sum of squares for 2 to 20 clusters was plotted (i.e. the elbow method), and k = 5 clusters was chosen to capture the major patterns. Five clusters showing the expression of all exposure-response genes across samples were generated using the k-means clustering function from R (k = 5, with the best of 50 random starts). A heatmap of 5 k-means gene clusters was generated using the Bioconductor R package, ComplexHeatmap [43], with hierarchical clustering (for each k-means cluster) with Euclidean distance metric was used for the genes, and Pearson correlation was used for the samples. All code for heatmap generation is included in S1 File.

### Analysis of genes within clusters

The pooled list of exposure-response genes significant at 1 and/or 5 days (556 genes) were used for the heatmap. Each of the gene lists for the k-means clusters were input separately into GSEA’s overlap calculation tool and Ingenuity Pathway Analysis (IPA) to determine enrichment and association with biological pathways and to identify predicted upstream regulators. The results from both GSEA and IPA canonical pathway enrichment tests were assimilated and summarized.

### Identification of exposure duration-dependent genes

To test for interaction of exposure (smoke versus sham) and exposure duration (1 versus 5 days), gene expression was modelled in a post-hoc test using exposure, exposure duration, and an interaction term between these variables for all significant exposure-response genes. Genes that had a significant interaction term (q<0.05), i.e., responded differently at 1 and 5 days, were reported as “exposure duration-dependent genes”. The code for this interaction test is included in S1 File.

### Comparison of gene expression responses between 1-day and 5-day exposures

Responses of gene expression to smoke exposure, genotype, or sex, as shown by β coefficients for 1 day and 5 days, were plotted and compared using the correlation test (cor.test) in R. Regression lines and confidence intervals were plotted using the R package, DescTools. The code for generating these plots is included in S1 File.

### Gene set analysis

Gene set analysis was adapted from the Gene Set Enrichment Analysis (GSEA) [44] method, using the signed -log(p value) as the test statistic [44, 45]; all genes, regardless of significance of the q values, were included in this analysis. The p value evaluates the strength of association with exposure and how well we can detect it, while the sign of the β coefficient was included to reflect the direction of change in response to the variable of interest. Briefly, the sum of test statistics for each gene in the gene set was divided by the square root of the number of genes in the gene set, creating an average score for the entire gene set, to produce the composite “GSA score” for the gene set. All code for the gene set testing is located in S2 File. The Canonical Pathways list (GSEA, Broad Institute, version 5.2) was used; the input genes were filtered to include only those with identical symbols between mouse and human in a case-insensitive manner. The file containing all Canonical Pathway gene sets tested is included in S3 File. The threshold for gene set significance was estimated empirically from 1000 random permutations of sample labels [46]. Significant gene sets were identified with a 5% FDR threshold [42]. The summary table of the results of permutation testing are included in S7 Table.

Selected gene set analysis (extracellular matrix biology gene sets from the literature):

Customized gene lists derived from Burgstaller et al. [47] was used to evaluate the association of exposure and extracellular matrix components. Sample-based permutation testing (n = 1000 permutations) was done to estimate the background level of association with the gene sets.

A full list of the gene sets used in this analysis are included in S4 File.

### Ingenuity pathway analysis

In order to get a more complete picture of the biological processes represented in the exposure-response genes identified, a second database of gene sets was used. IPA’s gene sets, which were derived from several databases and compiled from published literature, are distinct from GSEA’s Canonical Pathways list, which is derived from several databases compiling published literature. The networks and functional analyses were generated through the use of Ingenuity Pathways Analysis (version 2.3, https://www.qiagenbioinformatics.com/products/ingenuity-pathway-analysis/) [48].

### Accession code

The microarray data have been deposited into the Gene Expression Omnibus (http://www.ncbi.nlm.nih.gov/geo/) under series GSE109776.

## Results

### Effect of smoke exposure on airway inflammation

The numbers, ages and weights of mice are shown in Table 1. There was no difference between sham and smoked exposed mice in age or weight. Not surprisingly, the female mice have lower body weights than the males at the same age. The volume of BAL fluid used for each mouse was determined by the weight, so that the male and female concentrations of leukocytes and mediators can be compared.

**Table 1:** Description of WT and βENaC mice.

	Duration of exposure	Genotype	Exposure	n	Age (weeks)	Weight (g)
**Males**	1 day	WT	Sham	5	7.1 ± 0.2	22.4 ± 0.5
		WT	Smoke	5	7.2 ± 0.2	22.7 ± 1.7
		βENaC	Sham	6	7.0 ± 0.1	22.0 ± 0.8
		βENaC	Smoke	6	7.1 ± 0.2	23.1 ± 0.5
	1 day*	WT	Sham	5	6.9 ± 0.4	23.0 ± 0.8
		WT	Smoke	5	7.3 ± 0.5	22.3 ± 1.5
		βENaC	Sham	5	6.9 ± 0.4	20.6 ± 0.5
		βENaC	Smoke	4	7.3 ± 0.5	21.0 ± 1.1
	5 days	WT	Sham	7	6.2 ± 0.2	22.0 ± 0.7
		WT	Smoke	7	6.4 ± 0.1	19.5 ± 0.8
		βENaC	Sham	7	6.2 ± 0.2	20.6 ± 0.5
		βENaC	Smoke	7	6.4 ± 0.1	19.6 ± 0.9
**Females**	1 day	WT	Sham	5	7.0 ± 0.1	17.7 ± 0.9
		WT	Smoke	5	7.0 ± 0.2	16.4 ± 0.6
		βENaC	Sham	5	7.0 ± 0.1	18.0 ± 0.5
		βENaC	Smoke	5	7.0 ± 0.2	17.2 ± 0.7
	1 day*	WT	Sham	4	8.3 ± 0.6	18.5 ± 1.0
		WT	Smoke	5	7.6 ± 0.4	17.8 ± 0.6
		βENaC	Sham	4	7.9 ± 0.8	16.8 ± 1.4
		βENaC	Smoke	5	7.3 ± 0.5	18.0 ± 1.0
	5 days	WT	Sham	5	6.7 ± 0.4	18.2 ± 0.5
		WT	Smoke	5	6.4 ± 0.3	17.5 ± 0.3
		βENaC	Sham	5	6.7 ± 0.4	16.8 ± 0.5
		βENaC	Smoke	5	6.0 ± 0.4	15.8 ± 0.8

Age and weight are expressed as mean ± SEM.

*These 1-day exposed mice were used exclusively for analysis of gene expression by qPCR.

**Fig 1:**
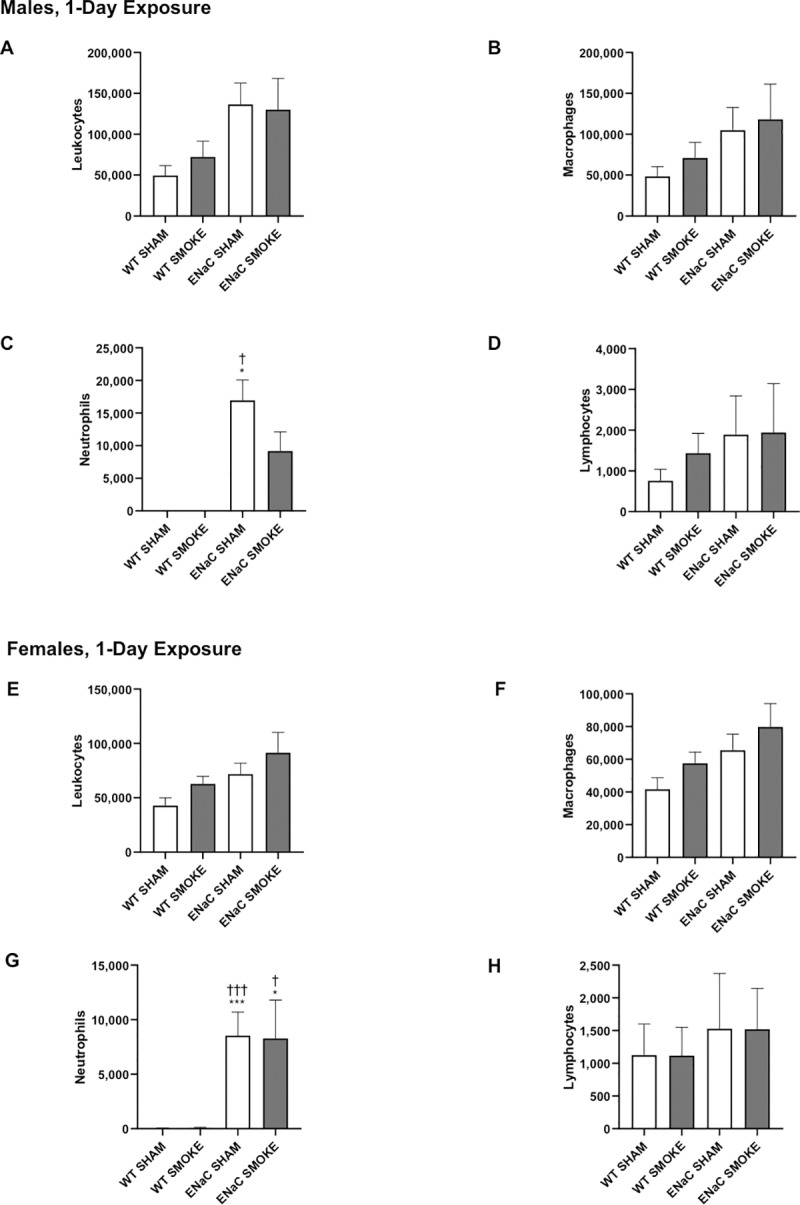
Effect of 1-day cigarette smoke exposure on BAL leukocyte numbers. The leukocyte counts in the BAL are described for males (A-D) and females (E-H) after 1-day sham or smoke exposure. A, E: leukocytes; B, F: macrophages; C, G: neutrophils; D, H: lymphocytes. Data are expressed as the total number of each leukocyte subtype in the BAL fluid. Analysis by ANOVA with Bonferroni’s post hoc test. Significance compared to (*) sham-exposed WT, (†) smoke-exposed WT, (‡) sham-exposed βENaC. Analysis by unpaired t-test: significance compared to (§) sham-exposed WT, (¶) smoke-exposed WT, (||) sham-exposed βENaC. Single symbols indicate p values < 0.05, double symbols indicate p values <0.01, and triple symbols indicate p values <0.001. Bars represent mean ± SEM.

**Fig 2:**
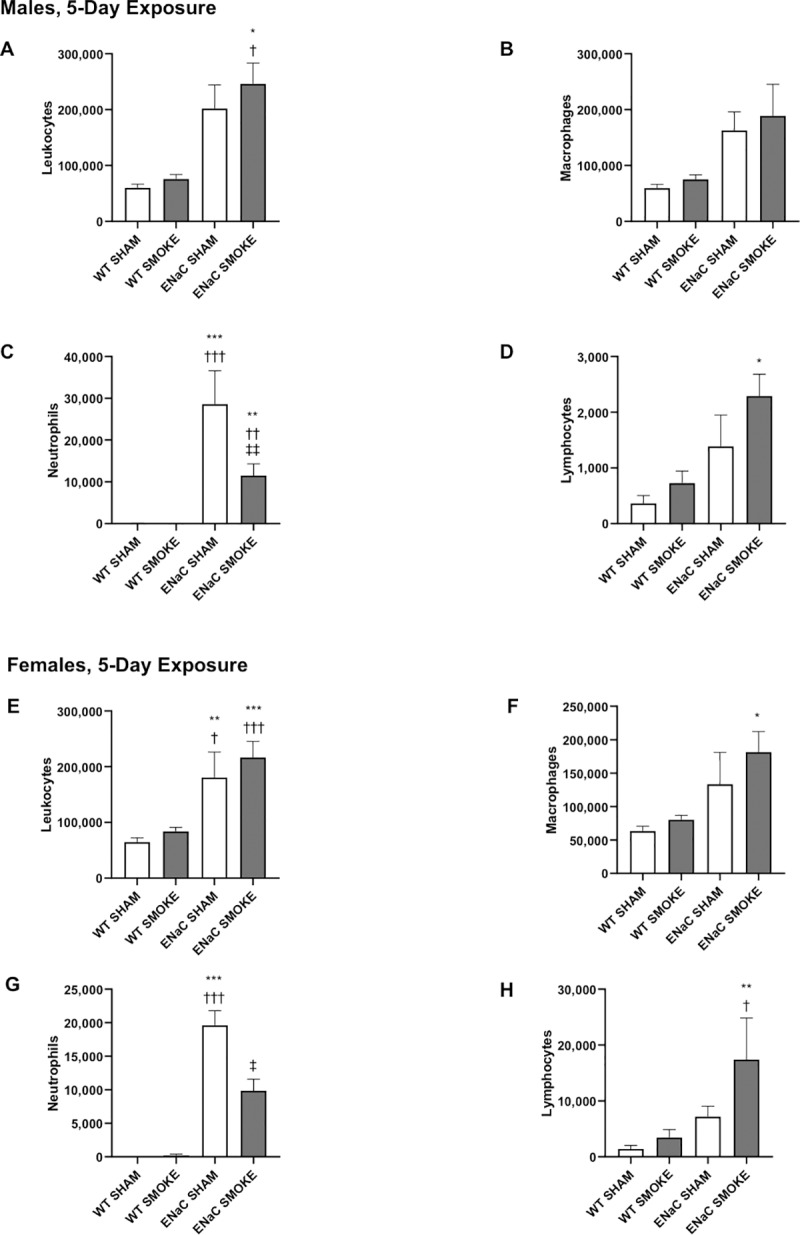
Effect of 5-day cigarette smoke exposure on BAL leukocyte numbers. The leukocyte counts in the BAL are described for males (A-D) and females (E-H) after 5-day sham or smoke exposure. A, E: leukocytes; B, F: macrophages; C, G: neutrophils; D, H: lymphocytes. Data are expressed as the total number of each leukocyte subtype in the BAL fluid. Analysis by ANOVA with Bonferroni’s post hoc test. Significance compared to (*) sham-exposed WT, (†) smoke-exposed WT, (‡) sham-exposed βENaC. Analysis by unpaired t-test: significance compared to (§) sham-exposed WT, (¶) smoke-exposed WT, (||) sham-exposed βENaC. Single symbols indicate p values < 0.05, double symbols indicate p values <0.01, and triple symbols indicate p values <0.001. Bars represent mean ± SEM.

Leukocytes were quantified in the BAL fluid collected from male and female WT and βENaC mice after 1-day (Fig 1) or 5-day (Fig 2) exposure to smoke. There was a trend toward more leukocytes in both the sham and smoke-exposed βENaC mice compared to WT mice that reached significance in mice studied at 5 days.

The number of BAL macrophages in mice exposed to smoke for 1 day did not significantly change with genotype or smoke exposure (Figs 1B and 1F). After 5 days, male βENaC mice exposed to cigarette smoke showed a significant increase in BAL macrophages, compared to sham-exposed WT males (Fig 2B). Female mice exposed to 5 days of smoke demonstrated a similar trend, though not significant (Fig 2F).

BAL neutrophils were increased in sham-exposed βENaC mice compared to WT mice at both 1 and 5 days (Figs 1C, 1G, 2C and 2G), consistent with previous observations in the βENaC genotype [30]. Interestingly, following 5 days of cigarette smoke exposure, the BAL of male and female βENaC mice contained fewer neutrophils compared to sham-exposed βENaC mice (Fig 2C and 2G). Neutrophil counts in male βENaC mice exposed to a single day of smoke tended to be less than in the sham-exposed controls, although the trend did not reach significance at this time (Fig 1C).

Neutrophils residing in the lungs (pulmonary tissue and airspace) were quantified by determining the number of CD45+ Ly6G+ cells in the lung digest using flow cytometry (Table 2). There was no significant difference due either to smoke compared sham exposure or to genotype after either 1- or 5-day exposures. However, both the WT and the ßENaC male lungs contained more neutrophils than female lungs after 1 day of cigarette smoke (Table 2).

**Table 2:** The numbers of neutrophils (CD45+, Ly6G+ cells) in the lung digests.

Duration of exposure	Genotype	Type of exposure	Females	Males
**1 day**	WT	Sham	8.23 ± 1.23 x 10^5^	16.6 ± 6.06 x 10^5^
		Smoke	6.04 ± 0.91 x 10^5^	23.2 ± 6.86 x 10^5^ *
	βENaC	Sham	8.56 ± 1.10 x 10^5^	19.5 ± 6.47 x 10^5^
		Smoke	7.77 ± 1.91 x 10^5^	28.0 ± 8.15 x 10^5^ *
				
**5 days**	WT	Sham	15.8 ± 7.75 x 10^5^	7.09 ± 2.92 x 10^5^
		Smoke	5.93 ± 1.65 x 10^5^	14.5 ± 10.7 x 10^5^
	βENaC	Sham	9.96 ± 2.34 x 10^5^	10.5 ± 4.06 x 10^5^
		Smoke	10.3 ± 2.75 x 10^5^	15.0 ± 7.92 x 10^5^

Lungs were subjected to enzymatic and mechanical digestion, and the cells were immunolabeled to mark neutrophils (CD45+, Ly6G+ cells). After 1-day exposure, male lungs contained more neutrophils than female lungs in both WT and βENaC genotypes. However, there was no significant effect of smoke compared to sham exposure in any group. Data are expressed as mean + SEM, the number in each group are described in Table 1.

*: significantly greater than similarly exposed female mice, p < 0.05 using unpaired t tests.

BAL lymphocytes were significantly elevated in βENaC males and females exposed to smoke for 5 days compared to WT mice, but not compared to sham-exposed βENaC mice (Fig 2D and 2H). No differences were observed in any group studied after 1 day (Fig 1D and 1H).

Sex differences in smoke-induced airway inflammation were assessed by comparing male and female mice in their response to cigarette smoke using an ANOVA with post hoc tests to compare sex, genotype and exposure (sham vs smoke). These comparisons showed that female βENaC mice exposed to smoke for 1 day had fewer BAL leukocytes than βENaC males. In addition, sham-exposed βENaC females had fewer BAL neutrophils than their male counterparts, which was significant at 1 day. Five days of smoke exposure induced a greater number of BAL lymphocytes in βENaC females compared to βENaC males.

Thus, taken together, these data suggest that the effect of smoke on leukocyte numbers and location depends on the genotype. WT mice demonstrated no effect of smoke compared to sham exposure on any leukocyte subpopulation, whereas smoke induced a decrease in lavageable neutrophils in the βENaC mice, particularly after 5 days. Because no differences were observed in the total number of neutrophils within the lungs using lung digests, this difference in lavageable neutrophils is more likely due to the increased adhesivity of neutrophils, rendering them less lavageable, rather than to cell death. Furthermore, both the βENaC transgene-induced phenotype and smoke were required for the increase in macrophages and lymphocytes. Importantly, these changes were not present after 1 day, but required 5 days of smoke exposure to manifest. These data suggest that profiling gene expression changes induced by smoke in both genotypes may prove fruitful in understanding the response of the lungs to smoke. Gene profiling may also answer questions about the rapidity of the antioxidant response in the lungs.

### Effects of smoke exposure on expression of inflammatory mediators and matrix metalloproteinases (MMPs)

The mRNA expression of select mediators and MMPs was measured in lung tissue after exposure to 1 or 5 days of smoke or sham air using PCR. mRNA expression was compared using ΔΔCt normalized to sham-exposed, WT 18S expression (Tables 3 and 4). Three chemokines, KC, MIP-2, and LIX, well recognized neutrophil chemoattractants, demonstrated significant changes. Both female and male βENaC mice exposed to sham air for 1 day expressed more KC, MIP-2, and LIX mRNA compared to WT sham- and smoke-exposed mice (Table 3), indicating that the increased chemokines were a result of the βENaC genotype. Interestingly, KC and MIP-2 expression decreased in female βENaC mice following a single day of smoke exposure when compared to sham-exposed βENaC females. KC expression increased in WT females exposed to 1 day of smoke compared to WT sham-exposed mice, and in fact this was one of very few instances in which smoke had a measurable effect in WT mice. MIP-2 and LIX expression was increased in smoke-exposed βENaC males compared to WT smoke- and sham-exposed males, but was not significantly altered in comparison to sham-exposed βENaC males.

**Table 3:** Expression of chemokine, cytokine and MMP mRNAs following 1-day exposure to cigarette smoke.

**Male mice**					
mRNA	WT sham	WT smoke	βENaC sham	βENaC smoke
KC	1.00 ± 0.37	0.90 ± 0.30	5.85 ± 1.06^†^^†^^,^**	3.72 ± 0.88
MIP-2	1.00 ± 0.32	1.31 ± 0.30	5.28 ± 0.50^†^^†^^†^^,^***	4.58 ± 0.50^†^^†^^,^***
LIX	1.00 ± 0.14	0.89 ± 0.31	9.51 ± 0.59^§^^§^^§^^,^^¶^^¶^	10.77 ± 3.72^†^^,^*
IFNγ	1.00 ± 0.51	2.40 ± 0.66	1.07 ± 0.17	0.82 ± 0.12
TNFα	1.00 ± 0.40	0.67 ± 0.20	1.87 ± 0.36	1.16 ± 0.20
IL-1β	1.00 ± 0.34	2.06 ± 1.46	0.81 ± 0.13	0.65 ± 0.18
IL-6	1.00 ± 0.29	0.83 ± 0.30	1.68 ± 0.35	4.57 ± 1.59
IL-10	1.00 ± 0.52	0.79 ± 0.20	0.58 ± 0.10	0.58 ± 0.15
MMP9	1.00 ± 0.34	1.66 ± 0.95	0.64 ± 0.15	0.49 ± 0.23
MMP12	1.00 ± 0.30	0.75 ± 0.19	7.09 ± 0.87^§^^§^^,^^¶^^¶^^¶^	7.46 ± 2.96
**Female mice**					
mRNA	WT sham	WT smoke	βENaC sham	βENaC smoke
KC	1.00 ± 0.25	2.40 ± 0.48^§^	12.83 ± 3.21^†^^†^^,^**	4.59 ± 1.60^‡^
MIP-2	1.00 ± 0.17	1.31 ± 0.12	13.97 ± 3.81^†^^†^^,^**	4.14 ± 1.33^‡^
LIX	1.00 ± 0.27	1.28 ± 0.44	7.83 ± 1.72^†^^,^**	4.13 ± 1.50
IFNγ	1.00 ± 0.29	2.45 ± 0.92	4.02 ± 1.35	1.57 ± 1.09
TNFα	1.00 ± 0.10	3.00 ± 0.47	3.98 ± 1.05*	2.52 ± 0.53
IL-1β	1.00 ± 0.35	6.74 ± 2.96	2.54 ± 0.38^§^	5.13 ± 3.38
IL-6	1.00 ± 0.34	5.17 ± 2.20	6.63 ± 1.87^§^	2.06 ± 0.56^||^
IL-10	1.00 ± 0.23	0.92 ± 0.20	0.44 ± 0.11	0.68 ± 0.14
MMP9	1.00 ± 0.30	5.05 ± 1.31^§^	1.21 ± 0.33^¶^	3.43 ± 1.85
MMP12	1.00 ± 0.32	3.95 ± 0.79^§^	22.54 ± 3.39^†^^†^^†^^,^***	5.43 ± 1.53^‡^^‡^^‡^^,^^§^

The ΔΔCT values were normalized to the sham-exposed WT 18S expression and then expressed as fold change following 1 day of sham or smoke exposure. Data are expressed as mean ± SEM, n = 4 or 5 mice, as described in Table 1. Analysis by ANOVA with Bonferroni’s post hoc test: significance compared to (*) sham-exposed WT

(†) smoke-exposed WT

(‡) sham-exposed βENaC. Analysis by unpaired t-test: significance compared to (§) sham-exposed WT

(¶) smoke-exposed WT

(||) sham-exposed βENaC. Single symbols indicate p values <0.05, double symbols indicate p values <0.01, and triple symbols indicate p values <0.001.

**Table 4:** Expression of chemokine, cytokine and MMP mRNAs following 5-day exposure to cigarette smoke.

**Male mice**				
mRNA	WT sham	WT smoke	βENaC sham	βENaC smoke
KC	1.00 ± 0.16	1.29 ± 0.18	2.11 ± 0.82	2.76 ± 0.77^§^
MIP-2	1.00 ± 0.13	1.40 ± 0.28	2.68 ± 0.60^§^	4.32 ± 1.14^†^^,^*
LIX	1.00 ± 0.41	1.22 ± 0.33	6.50 ± 3.31	5.01 ± 1.27
IFNγ	1.00 ± 0.10	1.57 ± 0.33	1.56 ± 0.52	2.94 ± 1.20
TNFα	1.00 ± 0.07	1.49 ± 0.25	2.08 ± 0.73	2.56 ± 0.48^§§^
IL-1β	1.00 ± 0.28	2.19 ± 0.91	1.25 ± 0.39	4.02 ± 2.84
IL-6	1.00 ± 0.25	2.03 ± 0.56	1.62 ± 0.85	2.82 ± 1.37
IL-10	1.00 ± 0.32	0.79 ± 0.25	0.66 ± 0.13	0.98 ± 0.31
MMP9	1.00 ± 0.41	0.82 ± 0.30	0.48 ± 0.21	1.05 ± 0.79
MMP12	1.00 ± 0.39	1.38 ± 0.66	7.26 ± 3.39	16.82 ± 7.82
**Female mice**				
mRNA	WT sham	WT smoke	βENaC sham	βENaC smoke
KC	1.00 ± 0.19	2.15 ± 0.67	4.29 ± 2.05	13.90 ± 7.15
MIP-2	1.00 ± 0.19	2.94 ± 0.84	5.32 ± 2.87	16.55 ± 6.39*
LIX	1.00 ± 0.20	1.42 ± 0.93	8.73 ± 3.13	7.94 ± 3.23^§,^^¶^
IFNγ	1.00 ± 0.36	1.56 ± 0.62	1.21 ± 0.19	1.50 ± 0.53
TNFα	1.00 ± 0.14	1.27 ± 0.27	2.59 ± 0.60^§^	2.44 ± 0.91
IL-1β	1.00 ± 0.38	1.99 ± 0.73	1.57 ± 0.36	3.37 ± 2.09
IL-6	1.00 ± 0.33	1.85 ± 0.82	3.18 ± 1.21	3.96 ± 2.73
IL-10	1.00 ± 0.33	0.83 ± 0.19	1.08 ± 0.16	1.16 ± 0.26
MMP9	1.00 ± 0.16	1.05 ± 0.20	0.96 ± 0.19	0.56 ± 0.20
MMP12	1.00 ± 0.32	2.20 ± 0.63	30.12 ± 3.05	73.56 ± 55.31

The ΔΔCT values were normalized to the sham-exposed WT 18S expression and then expressed as fold change following 5 days of sham or smoke exposure. Data are expressed as mean ± SEM, n = 4 or 5 mice, as described in Table 1. Analysis by ANOVA with Bonferroni’s post hoc test: significance compared to (*) sham-exposed WT

(†) smoke-exposed WT. Analysis by unpaired t-test: significance compared to (§) sham-exposed WT

(¶) smoke-exposed WT. Single symbols indicate p values <0.05, and double symbols indicate p values <0.01.

Following 5 days of smoke or sham exposure, no differences were observed in mRNA expression of KC and LIX in females (Table 4). MIP-2 expression was greater in female βENaC smoke-exposed mice compared to WT sham-exposed females. KC expression was higher in male βENaC mice exposed to 5 days of smoke compared to WT sham. MIP-2 expression was greater in sham-exposed βENaC males compared to WT sham, and in smoke-exposed male βENaC mice compared to both sham- and smoke-exposed WT mice. LIX expression increased in smoke-exposed βENaC male mice compared to WT males exposed to sham and smoke air.

The relative mRNA expression of 5 cytokines was also measured: TNFα, IFNγ, IL-1β, IL-6, and IL-10. Following 1 day of exposure, the lungs of βENaC sham-exposed females expressed increased levels of TNFα, IL-1 β, and IL-6 compared to WT sham females (Table 3). IL-6 expression was significantly decreased in βENaC females exposed to smoke compared to those exposed to sham air. There were no differences in IFNγ or IL-10 expression in the 1-day female groups. No change in any cytokine was observed in male mice between genotype or exposure.

Following 5 days of exposure, female βENaC mice exposed to sham air had increased expression of TNFα compared to their WT sham-exposed counterparts (Table 4). Smoke induced a small increase in TNFα in βENaC males compared to WT sham-exposed males. No differences were observed in the expression of IFNγ, IL-1β, IL-6, or IL-10 in the 5-day exposure group for either sex.

Measurement of MMP9 and MMP12 mRNA levels revealed interesting changes. Expression of both MMP9 and MMP12 increased in WT females after 1 day but not after 5 days of smoke exposure (Tables 3 and 4). βENaC sham-exposed females expressed higher levels of MMP12 than WT females exposed to sham or smoke. Interestingly, MMP12 expression was lower in βENaC females exposed to smoke. Male mice showed no change in MMP9 expression, but expression of MMP12 was increased in βENaC sham-exposed mice compared to both sham- and smoke-exposed WT mice.

Sex differences in inflammatory mediators and MMP expression were observed. A 1-day smoke exposure induced a significant increase in KC, MMP9 and MMP12 and a nearly significant increase in IL-1β and IL-6 that was not observed in male mice (Table 3). Female βENaC mice exposed to sham air expressed higher levels of KC, MIP-2, IL-6, and MMP12 mRNAs compared to male βENaC mice exposed to sham air studied at 1 day (Table 3). βENaC females exposed to 5 days of cigarette smoke had higher KC and MIP-2 expression than βENaC males with the same smoke exposure (Table 4).

Analysis of gene expression changes between 1- and 5-day exposures revealed interesting changes (Tables 3 and 4). MIP-2 expression significantly increased from 1 to 5 days of smoke exposure in female βENaC mice. A similar trend in MIP-2 expression was observed in WT female mice but did not reach significance. MMP9 expression decreased from 1 to 5 days of smoke exposure in WT females. A similar trend was observed in βENaC females, though it did not reach statistical significance. MMP12 expression in both male and female βENaC lungs appeared to increase between 1 and 5 days of smoke with a high degree of variability and did not achieve significance.

Thus, taken together, in female WT mice, smoke caused an increase in the mRNA expression of the chemokine KC and the two metalloproteinases, MMP9 and MMP12 after exposure for 1 day that was not observed in male mice or in either sex after 5 days. Sham-exposed female βENaC lungs expressed higher levels of many chemokines, cytokines and MMP12 than WT mice, and smoke resulted in a decrease in KC, MIP-2, IL-6 and MMP12. The only differences in male mice in the expression of the genes examined were due to genotype, and male mice showed no effect of smoke. Both the studies of leukocytes and of mediators suggest that the lungs rapidly upregulate protective mechanisms against the oxidant stress induced by inhalation of particulate and gaseous components of cigarette smoke. Gene profiling of lung tissue was performed to assess the rapidity and nature of these protective mechanisms, as well as to better understand other aspects of the lungs’ response.

Analysis of the protein expression of chemokines and cytokines in the BAL fluid using a multiplex ELISA did not reveal any significant effects of 1- or 5-day smoke exposure when measured 16 or 24 hours later in BAL fluid. Because BAL fluid represents a dilution of the epithelial cell lining fluid of 50–100 fold, low concentrations and small changes in expression are unlikely to be detected.

### Plasma levels of cotinine

In separate studies performed to assess the effect of smoke exposure on gene expression, plasma samples were obtained 4 hours after 1- or 5-day exposures, and the concentration of cotinine was measured. No cotinine was detected in any sample from sham-exposed mice (Table 5). Cotinine was present after 1- or 5-day exposures to cigarette smoke, and there was no difference in 1-day compared to 5-day exposures when each sex and genotype are compared individually. When values from males and females of both genotypes are pooled, 1-day exposures resulted in a higher plasma cotinine concentration than 5-day exposures (52.1 ± 4.9 ng/mL after 1-day exposure *vs*. 33.4 ± 5.4 ng/mL after exposure for 5 days).

**Table 5:** Concentration of cotinine in plasma (ng/mL plasma) measured by an ELISA.

	1-day exposure		5-day exposure	
Genotype	Sham	Smoke	Sham	Smoke
WT mice				
Male	None detected	44.8 ± 5.3	None detected	35.6 ± 9.2
Female	None detected	47.6 ± 3.8	None detected	31.0 ± 7.7
βENaC mice				
Male	None detected	52.1 ± 10.9	None detected	40.3 ± 3.7
Female	None detected	64.0 ± 4.1	None detected	27.2 ± 10.6

Cotinine concentration in the plasma samples show a clear distinction between smoke- and sham-exposed animals and are not significantly different with regard to sex or genotype. Data are expressed as mean ± SEM. N = 5 per group except n = 6 WT sham 5-day female mice, n = 4 βENaC sham 5-day female mice, and n = 4 WT smoke 5-day male mice.

### Exposure, genotype, and sex cause changes in hundreds of genes in the gene expression profile

In order to explore the variation within each duration of exposure and compare the responses, the samples from the 1-day and 5-day exposure durations were analyzed separately. To assess gene expression changes at 1 compared to 5 days of smoke exposure, the number of significant genes associated with exposure (smoke vs sham), genotype (WT vs βENaC), and sex were recorded (Table 6, S2 and S3 Tables).

**Table 6:** Differences in the number of genes influenced by each variable of interest.

	1 day	5 days
**Exposure**	330	347
**Genotype**	467	772
**Sex**	253	59

Smoke exposure, genotype, sex, and exposure duration all contribute important information to the gene expression profile. Only genes with a significant association (q<0.05) with the variable of interest and a fold change >+/-1.3 are recorded.

This analysis showed that after 1 and 5 days of smoke, exposure caused hundreds of genes to change significantly (330 and 347 genes, respectively, S4 and S5 Tables). Genotype has the greatest impact on the gene expression profile, showing the largest number of impacted genes after both 1 and 5 days (467 and 772 genes, respectively). The number of sex-response genes varies greatly, from 253 genes changed after 1 day of smoke to 59 genes changed after 5 days of smoke.

### The exposure-response gene profile clusters into five distinct clusters, which represent different biological pathways

**Fig 3:**
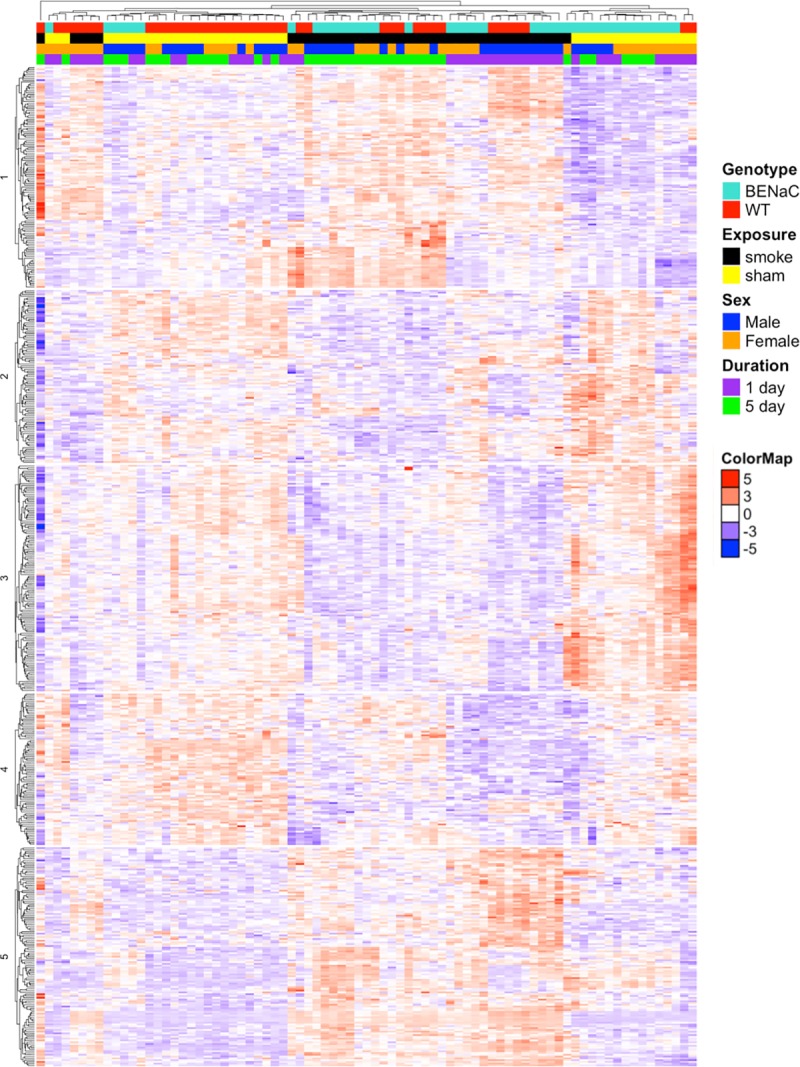
Differences in the gene expression profile between smoke and sham exposure. A heatmap of all samples showing the expression levels of the pooled list of exposure-response genes that were significantly associated with exposure after 1 and/or 5 days and with a fold change greater than +/-1.3. The color bar above the heatmap provides information about each sample. The samples subdivide into smoke- and sham-exposed samples, and then further subdivide by exposure duration (1 vs 5 days) in the smoke-exposed samples and by genotype (WT vs βENaC) in the sham-exposed animals. The genes were clustered into 5 clusters with distinct expression patterns using k-means clustering.

To visualize the patterns of gene expression changes associated with exposure, the normalized log2 intensities of the combined 556 exposure-response genes from 1 and 5 days (hereafter, the “pooled exposure-response genes”) were compiled and clustered using k-means and hierarchical clustering (Fig 3). The samples segregated first into smoke and sham, as expected, with two distinct groups of sham-exposed samples. Among the smoke-exposed samples, there was further grouping by exposure duration (1 day vs 5 days), which was not present in the sham-exposed samples. Among the sham-exposed samples, there was further grouping by genotype (WT vs βENaC). Genotype did not segregate within the smoke-exposed groups, and there was no segregation by sex in any group.

The 556 exposure-response genes clustered into five clusters with distinct expression patterns using the k-means algorithm. Three clusters (2, 3 and 4) contain genes that were downregulated in response to smoke, and two clusters (1 and 5) contain genes that were upregulated in response to smoke. The list of the genes in each cluster was then input into both GSEA and Ingenuity Pathway Analysis to understand the biological pathways represented. A summary of the Canonical Pathways that are significantly enriched in each cluster and how these genes respond to smoke exposure is presented in Table 7.

**Table 7:** Changes due to exposure at 1 and/or 5 days.

		B Coefficient for Exposure		Expression Change in	
Cluster	Key genes	1 day	5 days	Response to Smoke	Representative Pathways
1	Ces1g	2.07	2.61	Upregulated	Drug Metabolism by Cytochrome P450
	Ptgs2	1.37	0.64		Biological Oxidations
	1810010H24Rik	0.82	0.50		Nicotine Degradation II
	Slc4a1	0.71	1.89		Glutathione-mediated Detoxification
	Apol11b	0.43	1.56		Estrogen Biosynthesis
2	Pigr	-0.59	-0.01	Downregulated	Matrisome
	Slurp1	-0.63	-0.28		ECM Glycoproteins
	Plcb1	-0.74	-0.92		Chemokine Signaling
	Ighv12-3	-0.20	-1.08		ECM Regulators
	Spon2	-0.46	-1.22		Cytokine-Cytokine Receptor Interaction
	*Clca3*	1.15	-1.61		Collagen Formation
	*Adamts17*	0.61	-0.79		Focal Adhesion
	*Eln*	0.54	-0.39		ECM Organization
3				Downregulated	Immune System
	Serpinb10	-1.05	-0.81		Granulocytes Pathway
	Emr4	-1.12	-1.05		Interferon Signaling
	Ifitm6	-1.14	-0.87		Phagosome Formation
	Ccr3	-0.68	-0.91		Role of Pattern Recognition Receptors in Recognition of Bacteria and Viruses
					Leukocyte Extravasation Signaling
4	Slc10a5	-0.79	-0.08	Downregulated	
	Igkv4-80	-0.81	-1.02		No canonical pathways with
	Ccdc129	-1.15	-0.22		significant association
	Aplnr	-0.21	-0.74		
	Fabp1	-0.26	-1.13		
5	Cyp1a1	4.11	4.59	Upregulated	NRF2-mediated Oxidative Stress Response
	Slc7a11	3.65	1.69		Glutathione Biosynthesis
	Cyp1b1	3.16	3.01		Metabolism of Xenobiotics by Cytochrome P450
	Nqo1	2.87	2.64		Xenobiotic Metabolism Signaling
					Glutathione-mediated Detoxification

The clusters, depicted in Fig 1, are summarized. The key genes were identified as the top 3 genes up or downregulated by smoke; the top genes from 1 and 5 days were assimilated together into one list. Representative pathways were determined by significant enrichment in the Canonical Pathways from either the GSEA or IPA databases. Italicized genes are upregulated after 1 day and downregulated after 5 days of smoke exposure.

Clusters 1 and 5 contain genes upregulated in response to smoke. Genes in Cluster 5 are associated with cytoprotective processes to oxidative stress, including NRF2-mediated responses to oxidative stress and glutathione-mediated detoxification. The xenobiotic response, as mediated through cytochrome P450 enzymes, is also associated with this gene list. The genes in Cluster 1 are associated with processes such as nicotine degradation, drug metabolism by cytochrome P450 enzymes, glutathione-mediated detoxification, and estrogen biosynthesis.

Genes within Clusters 2 and 4 are downregulated in response to smoke. Cluster 2 genes are associated with the regulation and organization of the extracellular matrix (ECM). Pathways such as those involved in the matrisome, collagen formation, and ECM organization are enriched. A subset of the Cluster 2 genes are upregulated after 1 day of smoke exposure but downregulated by 5 days. Interestingly, Cluster 4 genes also appear to have different responses to exposure duration in the smoke-exposed animals: they are downregulated to different magnitudes after 1 compared to 5 days of smoke. However, Cluster 4 genes are not significantly associated with any canonical pathways.

The downregulated Cluster 3 genes are enriched for biological pathways encompassing many aspects of the immune system response, such as granulocyte, monocyte, and B lymphocyte pathways. Immune responses including the Fc gamma receptor-mediated phagocytosis in macrophages and monocytes, phagosome formation, and the role of pattern recognition receptors in recognition of bacteria and viruses are also associated with the gene list from Cluster 3.

### A minority of exposure-response genes are also influenced by genotype and/or sex independently

**Fig 4:**
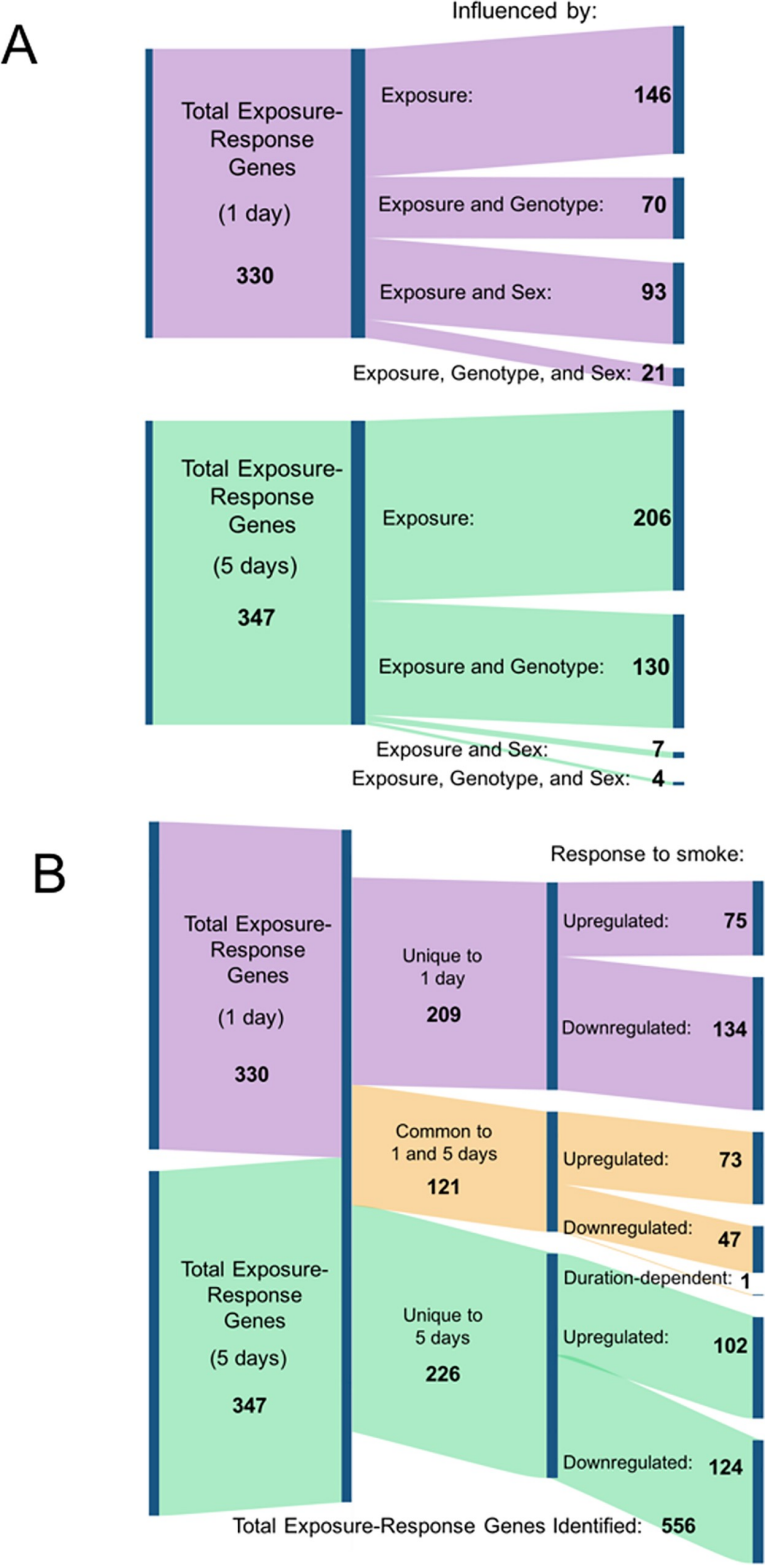
Breakdown of exposure-response genes by exposure duration and response to smoke. The Sankey diagram breaks down the genes in a hierarchical manner, and the area of the shape is proportional to the number of genes represented. Purple indicates results from 1 day of exposure; green indicates results from 5 days of exposure. (A): Sankey diagram showing the proportion of exposure-response genes additionally affected by genotype and/or sex: 56% of the exposure-response genes at 1 day and 41% of the genes at 5 days are further modulated by genotype and/or sex. At 1 day, 70 of the exposure-response genes were also influenced by genotype and 93 genes were also influenced by sex, while 21 of the exposure-response genes were influenced independently by exposure, genotype, and sex. After 5 days, 130 of the exposure-response genes were also influenced by genotype and 7 genes were also influenced by sex. Four genes were influenced independently by exposure, genotype, and sex. (B): Sankey diagram showing the proportion of exposure-response genes that are unique to each exposure duration (green and purple) compared to the proportion that responds significantly after both 1 and 5 days (orange). 556 total exposure-response genes were identified at 1 and/or 5 days of smoke exposure. At 1 day, 330 exposure-genes were identified. 209 of these genes were uniquely significantly associated with the 1 day exposure, while 121 genes were also identified as significantly associated after 5 days. After 5 days, 347 exposure-genes were identified, 226 of which were uniquely significantly associated with 5 days of exposure. Diagrams created using SankeyMATIC.

In order to understand the changes due to smoke in these exposure-response genes, it is important to understand whether the baseline expression level in sham-exposed mice is different between sexes and/or genotypes. We hypothesized that there are genotype- and sex-specific effects occurring during smoke exposure. Overall, there is no difference resulting from smoke exposure in males compared to females or in βENaC mice compared to WT mice. Our analysis showed no dependent relationship between smoke exposure and either genotype or sex. However, many independent effects were identified (Fig 4A), suggesting the effects of exposure, genotype, and sex on gene expression are largely additive. Many of the exposure-response genes are also influenced by genotype and/or sex; the changes in gene expression from all variables together make up the total change in the gene’s expression compared to wildtype sham-exposed mice. For 56% of the exposure-response genes at 1 day and 41% of the exposure-response genes at 5 days, the gene expression is changed significantly by both exposure and at least one other variable, independently. For example, 93 genes were associated with both exposure and sex after 1 day of smoke exposure (Fig 4A). Therefore, while we did not find many significant dependent relationships between exposure and either genotype or sex, the expression of many exposure-response genes is further influenced by genotype and sex, independently.

### Changes in the exposure response after 1 compared to 5 days of smoke

We next asked whether the exposure-response gene expression profiles at 1 and 5 days were different by assessing how many of the exposure-response genes were uniquely responsive at each exposure duration and how many were commonly significant after both 1 and 5 days of exposure. Although the number of changed genes is similar between 1 and 5 days, only 121 of these are changed after both 1 and 5 days. Of the 556 total unique genes that changed in response to exposure, the majority of the exposure-response genes (435 genes, 78%) have a specific, duration-dependent response (Fig 4B).

**Fig 5:**
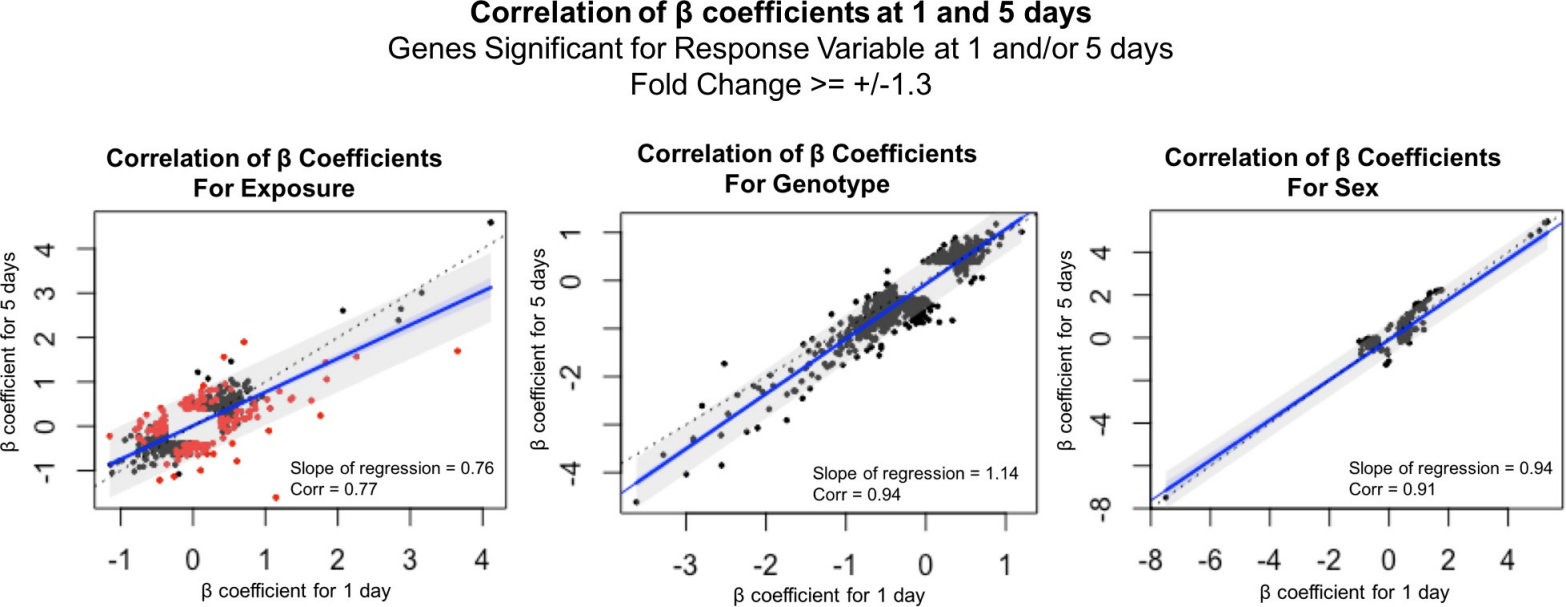
Correlation between 1- and 5-day β coefficients in significant response genes for each variable. The coefficient of correlation (“Corr”) is listed in the bottom right corner of the graph and reflects the tightness of fit of the observed β coefficients to a linear pattern. The slope of the best-fit regression line (“Slope of regression”, blue line) is also reported in the bottom right corner of the graph; the amount of deviation from the slope of the unity line (black dotted line) shows the amount of exposure-duration dependent response observed. The 95% confidence intervals for the regression line are outlined in blue, and the 95% prediction interval is outlined in grey. (A): Correlation of the exposure effects on expression in exposure-response genes at 1 and 5 days. Red dots indicate those genes which have significantly different responses at 1 and 5 days, as indicated by an interaction test. (B): Correlation of the genotype effects on expression in genotype-response genes at 1 and 5 days. (C): Correlation of the sex effects on expression in sex-response genes at 1 and 5 days.

Although there are specific genes that have a duration-dependent response, overall, the exposure response at 1 and 5 days is similar. To further compare the gene expression response to smoke exposure at these durations, we also looked at the correlation of β coefficients within the pooled response genes (Fig 5). Genotype- and sex-response genes have greater correlations (0.94 and 0.91, respectively) and slopes very close to one when comparing exposure durations, showing that the impact of smoke is approximately the same after a single exposure as after repeated exposures for 5 days (Fig 5). Exposure has the greatest variability of response between these exposure durations. The correlation coefficient of 0.77 and slope of 0.76 shows that the responses after 1 and 5 days of exposure are generally similar. However, some genes show different behavior that is exposure duration-dependent, as evidenced by a significant interaction between exposure and exposure duration (red dots in Fig 5A). We hypothesized that these genes exhibiting exposure duration-dependent behavior reflected important differences in the lung’s response after the first compared to repeated exposures to cigarette smoke.

### Gene sets describing the function of duration-dependent exposure-response genes include ECM and oxidative stress pathways

In fact, 165 exposure-response genes had significantly different responses at each exposure duration (S6 Table; red dots, Fig 5A). An overlap analysis was performed using this gene list and the GSEA’s Canonical Pathways list to determine if these genes represented any functional pathways that respond differently between 1 and 5 days of exposure (Table 8). Several of the associated gene sets fell into two categories: oxidation/conjugation of glutathione and regulation of the extracellular matrix (ECM). The duration-dependent exposure-response gene list had the most significant overlap with the NABA Matrisome gene set; 21 of the 165 duration-dependent exposure-response genes (q = 4.15e^-10^). The second most highly enriched gene set was the Reactome Biological Oxidations list, with 8 genes overlapping (q = 2.04e^-6^). These results suggest a role for processes regulating the oxidation/conjugation of glutathione and aspects of ECM biology, including the matrix structure and the attachments of cells to the matrix, in the modulation of the lung’s response to cigarette smoke.

**Table 8:** Gene sets enriched in the exposure-response genes with significantly different behavior at 1 compared to 5 days.

Gene Set Name	# Genes in Gene Set	# Genes in Overlap	FDR q-value
NABA_MATRISOME	1028	21	4.15E-10
REACTOME_BIOLOGICAL_OXIDATIONS	139	8	2.40E-06
NABA_CORE_MATRISOME	275	9	2.24E-05
REACTOME_GLUTATHIONE_CONJUGATION	23	4	1.25E-04
NABA_MATRISOME_ASSOCIATED	753	12	1.67E-04
REACTOME_NCAM_SIGNALING_FOR_NEURITE_OUT_GROWTH	64	5	1.67E-04
NABA_ECM_GLYCOPROTEINS	196	7	1.73E-04
REACTOME_AMINO_ACID_TRANSPORT_ACROSS_THE_PLASMA_MEMBRANE	31	4	2.19E-04
REACTOME_NCAM1_INTERACTIONS	39	4	5.00E-04
REACTOME_AXON_GUIDANCE	251	7	6.15E-04
REACTOME_TRANSPORT_OF_INORGANIC_CATIONS_ANIONS_AND_AMINO_ACIDS_OLIGOPEPTIDES	94	5	6.15E-04
REACTOME_AMINO_ACID_AND_OLIGOPEPTIDE_SLC_TRANSPORTERS	49	4	9.47E-04
KEGG_GLUTATHIONE_METABOLISM	50	4	9.48E-04
REACTOME_DEVELOPMENTAL_BIOLOGY	396	8	9.75E-04
REACTOME_TRANSMEMBRANE_TRANSPORT_OF_SMALL_MOLECULES	413	8	1.23E-03
REACTOME_SULFUR_AMINO_ACID_METABOLISM	24	3	2.62E-03
REACTOME_PHASE1_FUNCTIONALIZATION_OF_COMPOUNDS	70	4	2.62E-03
REACTOME_PHASE_II_CONJUGATION	70	4	2.62E-03
REACTOME_SLC_MEDIATED_TRANSMEMBRANE_TRANSPORT	241	6	3.03E-03
KEGG_ECM_RECEPTOR_INTERACTION	84	4	4.82E-03
REACTOME_METABOLISM_OF_AMINO_ACIDS_AND_DERIVATIVES	200	5	1.17E-02
KEGG_FOCAL_ADHESION	201	5	1.17E-02
REACTOME_ETHANOL_OXIDATION	10	2	1.63E-02
NABA_SECRETED_FACTORS	344	6	1.63E-02
REACTOME_SIGNALING_BY_PDGF	122	4	1.63E-02
NABA_ECM_REGULATORS	238	5	2.14E-02
KEGG_ARACHIDONIC_ACID_METABOLISM	58	3	2.29E-02
REACTOME_COLLAGEN_FORMATION	58	3	2.29E-02
KEGG_CIRCADIAN_RHYTHM_MAMMAL	13	2	2.37E-02
REACTOME_METABOLISM_OF_PORPHYRINS	14	2	2.67E-02
PID_INTEGRIN1_PATHWAY	66	3	3.02E-02
REACTOME_GPCR_LIGAND_BINDING	408	6	3.06E-02
PID_CIRCADIAN_PATHWAY	16	2	3.19E-02
KEGG_METABOLISM_OF_XENOBIOTICS_BY_CYTOCHROME_P450	70	3	3.27E-02
KEGG_DRUG_METABOLISM_CYTOCHROME_P450	72	3	3.45E-02
REACTOME_FATTY_ACID_TRIACYLGLYCEROL_AND_KETONE_BODY_METABOLISM	168	4	3.76E-02
PID_S1P_S1P1_PATHWAY	21	2	4.94E-02
KEGG_SMALL_CELL_LUNG_CANCER	84	3	4.96E-02

An overlap analysis performed by GSEA comparing the list of genes with significantly different behavior at 1 compared to 5 days of smoke exposure, as defined by a significant interaction effect between exposure and exposure duration (red dots in Fig 5A) to the Canonical Pathways gene set list. These genes overlap most significantly with several oxidative response gene sets, as well as those regulating ECM biology.

### The top gene sets significantly associated with both 1- and 5-day exposure responses represent xenobiotic and antioxidant response pathways

We next asked whether pathways involved in similar biological processes were becoming further stimulated or repressed over increasing exposure duration. After 1 day of exposure, 261 gene sets were significantly associated with the exposure response (S8 Table). After 5 days of exposure, 412 gene sets were significantly associated with the exposure response (S9 Table).

Xenobiotic responses, often through cytochrome P450, and antioxidant responses, often through the pathways of glutathione metabolism, are the most highly associated and are increased after both 1 and 5 days of exposure (S8 and S9 Tables). These biological pathways have been identified in several previous studies as highly responsive to cigarette smoke [28, 49, 50].

### A subset of exposure-associated gene sets respond differently to smoke at 1 compared to 5 days of exposure

Gene sets that are discordant by exposure duration change their direction of response to cigarette smoke between the first exposure and 5 days of exposure. The discordant exposure-associated gene sets provide some insight into how the lung modulates its response to cigarette smoke between the first exposure and 5 days of repeated exposures. The exposure-associated gene sets that were downregulated at 1 day but upregulated due to smoke after 5 days (Table 9) include both oxidative phosphorylation and the TCA cycle/respiratory electron transport. Moreover, the gene sets that are first upregulated but become downregulated in response to smoke after 5 days primarily include processes regulating the ECM. We therefore hypothesize that differential regulation of oxidative phosphorylation and ECM biology are among the processes changed by the lungs that account for the differential exposure response between the 1-day and 5-day durations.

**Table 9:** Gene sets that changed significantly, either similarly or differently, in response to smoke after both 1 and 5 days.

Direction of Response at 1 day	Direction of Response at 5 days	Gene Set Name	GSA Score at 1 day	GSA Score at 5 days
**Up**	**Down**	REACTOME_SIGNALING_BY_PDGF	21.00	-12.64
		NABA_BASEMENT_MEMBRANES	17.37	-17.54
		REACTOME_DEVELOPMENTAL_BIOLOGY	16.53	-18.85
		PID_FRA_PATHWAY	14.87	-7.31
		PID_ANGIOPOIETIN_RECEPTOR_PATHWAY	14.56	-10.29
		KEGG_MAPK_SIGNALING_PATHWAY	13.93	-9.64
		KEGG_MELANOMA	13.90	-7.44
		KEGG_FOCAL_ADHESION	13.55	-18.05
		REACTOME_HEMOSTASIS	13.18	-19.31
		NABA_ECM_GLYCOPROTEINS	12.79	-23.35
		PID_FAK_PATHWAY	12.21	-10.95
		PID_FOXM1_PATHWAY	12.10	-7.37
		REACTOME_AXON_GUIDANCE	11.98	-24.35
		NABA_CORE_MATRISOME	11.91	-27.24
		NABA_MATRISOME	11.28	-30.69
		PID_ER_NONGENOMIC_PATHWAY	11.08	-8.01
		PID_INTEGRIN1_PATHWAY	11.01	-22.65
		NABA_ECM_REGULATORS	10.81	-7.97
		PID_FGF_PATHWAY	10.47	-9.16
		PID_AVB3_INTEGRIN_PATHWAY	10.37	-16.30
		KEGG_GNRH_SIGNALING_PATHWAY	10.29	-8.97
		PID_NETRIN_PATHWAY	10.24	-11.38
**Down**	**Up**	KEGG_OXIDATIVE_PHOSPHORYLATION	-11.29	9.21
		REACTOME_RESPIRATORY_ELECTRON_TRANSPORT_ATP_SYNTHESIS_BY_CHEMIOSMOTIC_COUPLING_AND_HEAT_PRODUCTION_BY_UNCOUPLING_PROTEINS_	-12.28	11.23
		REACTOME_TCA_CYCLE_AND_RESPIRATORY_ELECTRON_TRANSPORT	-12.55	19.77
		REACTOME_RESPIRATORY_ELECTRON_TRANSPORT	-12.72	10.62
		KEGG_PROPANOATE_METABOLISM	-14.67	14.93
**Down**	**Down**	BIOCARTA_THELPER_PATHWAY	-10.13	-10.94
		REACTOME_SIGNAL_REGULATORY_PROTEIN_SIRP_FAMILY_INTERACTIONS	-10.53	-7.85
		BIOCARTA_TCYTOTOXIC_PATHWAY	-10.67	-10.58
		KEGG_INTESTINAL_IMMUNE_NETWORK_FOR_IGA_PRODUCTION	-11.00	-12.56
		REACTOME_BETA_DEFENSINS	-11.20	-8.38
		SA_MMP_CYTOKINE_CONNECTION	-11.65	-10.67
		REACTOME_IMMUNOREGULATORY_INTERACTIONS_BETWEEN_A_LYMPHOID_AND_A_NON_LYMPHOID_CELL	-12.21	-16.84
		KEGG_HEMATOPOIETIC_CELL_LINEAGE	-12.28	-13.15
		REACTOME_INTERFERON_GAMMA_SIGNALING	-12.58	-17.00
		BIOCARTA_BLYMPHOCYTE_PATHWAY	-13.37	-8.95
		BIOCARTA_GRANULOCYTES_PATHWAY	-13.84	-12.43
		KEGG_CELL_ADHESION_MOLECULES_CAMS	-14.53	-28.80
		BIOCARTA_LYM_PATHWAY	-18.38	-19.42
		BIOCARTA_MONOCYTE_PATHWAY	-19.68	-17.23
**Up**	**Up**	KEGG_METABOLISM_OF_XENOBIOTICS_BY_CYTOCHROME_P450	57.33	65.12
		REACTOME_GLUTATHIONE_CONJUGATION	47.64	45.07
		REACTOME_BIOLOGICAL_OXIDATIONS	45.90	49.79
		KEGG_DRUG_METABOLISM_CYTOCHROME_P450	42.73	52.15
		REACTOME_PHASE_II_CONJUGATION	37.08	37.77
		KEGG_GLUTATHIONE_METABOLISM	31.17	38.85
		REACTOME_PHASE1_FUNCTIONALIZATION_OF_COMPOUNDS	27.65	32.24
		BIOCARTA_P53HYPOXIA_PATHWAY	25.49	15.44
		REACTOME_XENOBIOTICS	24.97	32.03
		REACTOME_ETHANOL_OXIDATION	23.67	25.80
		REACTOME_METABOLISM_OF_PORPHYRINS	23.08	32.36
		KEGG_STEROID_HORMONE_BIOSYNTHESIS	23.03	22.06
		REACTOME_PPARA_ACTIVATES_GENE_EXPRESSION	22.36	16.64
		PID_TAP63_PATHWAY	21.99	15.78
		KEGG_TYROSINE_METABOLISM	21.23	23.40
		REACTOME_CYTOCHROME_P450_ARRANGED_BY_SUBSTRATE_TYPE	20.12	22.09
		REACTOME_ENDOGENOUS_STEROLS	19.90	16.65
		REACTOME_SULFUR_AMINO_ACID_METABOLISM	19.67	19.55
		REACTOME_PI3K_EVENTS_IN_ERBB2_SIGNALING	19.29	7.25
		KEGG_PORPHYRIN_AND_CHLOROPHYLL_METABOLISM	17.87	26.07
		REACTOME_GLUCURONIDATION	15.99	13.40
		PID_S1P_S1P1_PATHWAY	15.29	7.21
		KEGG_TRYPTOPHAN_METABOLISM	15.14	28.38
		REACTOME_BMAL1_CLOCK_NPAS2_ACTIVATES_CIRCADIAN_EXPRESSION	14.94	12.06
		REACTOME_CIRCADIAN_CLOCK	14.83	12.37
		REACTOME_REGULATION_OF_ORNITHINE_DECARBOXYLASE_ODC	14.41	12.16
		PID_DELTA_NP63_PATHWAY	13.82	9.73
		KEGG_PHENYLALANINE_METABOLISM	13.81	17.89
		KEGG_ABC_TRANSPORTERS	13.23	11.75
		KEGG_RETINOL_METABOLISM	13.18	20.13
		REACTOME_ANTIGEN_PROCESSING_UBIQUITINATION_PROTEASOME_DEGRADATION	13.16	8.98
		REACTOME_ABC_FAMILY_PROTEINS_MEDIATED_TRANSPORT	13.04	9.80
		PID_P38_GAMMA_DELTA_PATHWAY	13.03	7.66
		KEGG_REGULATION_OF_AUTOPHAGY	12.98	12.56
		KEGG_ARACHIDONIC_ACID_METABOLISM	12.93	12.42
		REACTOME_CYCLIN_E_ASSOCIATED_EVENTS_DURING_G1_S_TRANSITION_	12.86	9.91
		KEGG_NITROGEN_METABOLISM	12.84	7.50
		KEGG_INSULIN_SIGNALING_PATHWAY	12.59	8.95
		PID_RXR_VDR_PATHWAY	12.40	10.87
		REACTOME_P53_DEPENDENT_G1_DNA_DAMAGE_RESPONSE	11.81	8.47
		REACTOME_PKB_MEDIATED_EVENTS	11.67	9.18
		PID_HDAC_CLASSIII_PATHWAY	11.37	8.25
		REACTOME_SCFSKP2_MEDIATED_DEGRADATION_OF_P27_P21	11.26	7.75
		PID_HIF1_TFPATHWAY	11.25	12.95
		BIOCARTA_HIF_PATHWAY	11.13	8.67
		REACTOME_FATTY_ACID_TRIACYLGLYCEROL_AND_KETONE_BODY_METABOLISM	11.00	18.72
		BIOCARTA_NUCLEARRS_PATHWAY	10.85	9.05
		REACTOME_FORMATION_OF_INCISION_COMPLEX_IN_GG_NER	10.66	8.17
		REACTOME_METABOLISM_OF_AMINO_ACIDS_AND_DERIVATIVES	10.55	26.20
		KEGG_GLYCOLYSIS_GLUCONEOGENESIS	10.24	24.01
		REACTOME_DOWNREGULATION_OF_ERBB2_ERBB3_SIGNALING	10.04	7.24

The gene sets which are upregulated after 1 day and downregulated after 5 days of smoke involve mostly ECM-related processes. Those gene sets which are downregulated after 1 day and upregulated after 5 days of smoke involve primarily electron transport and oxidative phosphorylation pathways. The gene sets which are downregulated after both 1 and 5 days are exclusively associated with the immune response. The gene sets which are upregulated after both 1 and 5 days primarily describe metabolism of small molecules. Red highlighting: upregulated gene sets. Blue highlighting: downregulated gene sets.

Gene sets that responded similarly after both 1 day and 5 days of smoke exposure are also listed in Table 9. The gene sets that were downregulated after both 1 and 5 days of exposure are exclusively gene sets involved in immune processes. Among the gene sets that were upregulated in response to smoke after both 1 and 5 days, oxidative processes are the most numerous, although gene sets representing immune processes, changes in metabolism and energy sources, transcription factors, and hypoxia are also present. These results provide insight into the lung’s response to smoke exposure which do not appear to be differentially modulated between these two acute exposure durations.

### A more comprehensive look at ECM gene sets associated with exposure shows upregulation after 1 day and downregulation after 5 days of smoke

ECM biology regulation was significantly associated with smoke in the gene set association analyses and is recognized as important in cigarette-induced lung injury. In a recent review [47], Burgstaller and colleagues have carefully characterized the proteins involved in the ECM of the lung through mass spectrometric methods and provided the genes encoding them [47]. Since this comprehensive list of genes has not yet been added to the GSEA database, we created custom gene set lists derived from this publication and tested them for association with exposure in our dataset. The gene sets derived from this composite list of all ECM proteins, as well as the subset containing only glycoproteins, were significantly associated with exposure at both 1 and 5 days. Furthermore, these gene sets behaved discordantly with exposure duration: both the composite list of ECM components and the glycoproteins subset were upregulated after 1 day but downregulated after 5 days of smoke (Table 10). Interestingly, the data show that ECM gene expression was strongly associated with sex, and that expression of ECM genes was increased in male compared to female lung tissue. The few differences seen when genotypes are compared are small and inconsistent.

**Table 10:** Significant association of exposure-dependent changes in ECM gene sets from recent literature.

	GSA Score for:					
Pathway Name:	Exposure		Genotype		Sex	
	1 day	5 days	1 day	5 days	1 day	5 days
ECM_ALL_COMPONENTS_ERS_REVIEW_2017_EICKELBERG	**11.78**	**-31.69**	**-17.71**	3.03	**62.47**	**37.01**
ECM_COLLAGENS_ERS_REVIEW_2017_EICKELBERG	5.59	**-16.33**	**-12.88**	-0.09	**36.07**	**21.14**
ECM_PROTEOGLYCANS_ERS_REVIEW_2017_EICKELBERG	-1.29	-9.21	-8.23	2.20	**12.40**	7.08
ECM_GLYCOPROTEINS_ERS_REVIEW_2017_EICKELBERG	**11.83**	**-25.72**	-11.03	2.79	**51.05**	**30.48**

GSA scores for gene sets derived from a recent review [47]. Bold text indicates that the association was significant at a q<0.05 threshold.

### The impacts of genotype and sex on gene expression occur independently from exposure in acute exposure responses

The impacts of sex and genotype on the gene expression profile of the lungs are significant, as shown by the hundreds of genes and gene sets significantly associated with genotype and sex (Table 6 and S10–S13 Tables). Furthermore, sex and genotype explain significant variation in expression (S14 Table).

Additionally, the genotype-response genes significantly overlap with published lists of DEGs [35] for βENaC compared with WT mice for both the 1-day and 5-day genotype-response gene lists (p = 3.3e^-71^ and p = 1.5e^-74^, respectively), confirming that the experimental and analytical methods are robust (S1 Table). Our genotype-response gene lists also include many additional genes showing a significant difference between βENaC and WT mice with a fold change of at least 1.3.

Although large differences in the numbers of sex- and genotype-response genes at 1 day compared to 5 days of exposure were identified (Table 6), the β coefficients of these genes for 1 day compared to 5 days are significantly correlated (Fig 5). Individual genes may be responding in an exposure duration-dependent manner, however, there is no evidence for exposure duration-dependent sex or genotype differences in the gene expression profile at these acute exposure lengths. Taken together with the lack of dependent interactions with exposure at the individual gene level, these data suggest that genotype- and sex-associated changes occur independently of smoke exposure and of exposure duration.

### A comparison of the acute exposure responses with a published study of chronic smoke exposure in mice

The exposure-response genes identified after 1 and 5 days of smoke represent the lung’s response to a single and five consecutive daily doses of smoke. In order to explore how these responses compared to gene expression in the lung in an established environment of chronic smoke exposure, we compared these gene lists to DEGs identified in WT mice after six months of smoke exposure [51] by testing the overlap of these lists. Miller and colleagues identified 111 genes that were differentially expressed in smoke- compared to sham-exposed WT mice. There was significant overlap between this list of DEGs and our results. Of the exposure-response genes identified in our study, 26 of the 1-day genes (p = 2e-24) and 40 of the 5-day genes (p = 8.6e-45) were also identified after 6 months of smoke exposure by Miller and colleagues.

## Discussion

Understanding how the lungs cope with cigarette smoke following a single exposure and upon repeated exposures provides information about pathways and processes underlying host defense that is likely to be useful in understanding the development of chronic lung disease. Cigarette smoke is thought to act initially through the generation of lung cell damage due to oxidants present in the gaseous and particulate phases that initiate host defense. Our study tested the hypothesis that the pulmonary response to cigarette smoke, as measured by the immune response and the expression of exposure-response genes is rapid and changes over the short duration of 5 days.

Our studies sought to characterize the cellular immune response following 1 day and 5 days of smoke exposure. In WT mice, cigarette smoke did not recruit leukocytes to the lung parenchyma or the airspace. Smoke did induce an increase in the mRNA expression, as measured by PCR, of the chemokine KC and the two metalloproteinases, MMP9 and MMP12 after 1-day exposure of female mice that was not observed in male mice or in either sex after 5 days (Tables 3 and 4). βENaC mice had more airspace leukocytes compared to their WT counterparts following either sham or smoke exposure, confirming their phenotype as previously reported [31–33, 37]. Sham-exposed βENaC mice expressed higher levels of many mediators, females in particular expressing higher levels of chemokines, cytokines and MMP12 than WT mice. Thus, the βENaC genotype is responsible for the greater number of airspace immune cells and increased expression of cytokine mRNAs. Surprisingly, after 5 days, smoke induced a decrease in lavageable neutrophils in βENaC mice. Because lung digests showed no difference in the total number of neutrophils within the lung tissue, we suggest that the smoke-induced decrease in lavageable neutrophils is more likely due to activation of neutrophils leading to increased adhesivity. This increased adhesion to alveolar walls then renders them less lavageable, decreasing their concentration in the BAL. Since there was no loss of total neutrophils in the lungs, neutrophil death or increased turnover appears less likely. Furthermore, smoke resulted in less expression of KC, MIP-2, IL-6 and MMP12 mRNAs, as measured by PCR, compared to sham exposure in this genotype, suggesting that lungs that are already inflamed may be better able to respond to the additional burden of cigarette smoke very rapidly by downregulation of inflammatory responses. However, the increase in macrophages and lymphocytes required smoke exposure together with the βENaC transgene-induced phenotype. Importantly, these changes were not present after 1 day, but required 5 days of smoke exposure to manifest. These differences between critically important aspects of the inflammatory and immune response in βENaC compared to WT mice suggest that the lung microenvironment critically affects the response to inhaled cigarette smoke.

Thus, many cellular events were occurring that resulted in complex changes in leukocyte kinetics and that were likely to result in protection of lung parenchymal and immune cells from the oxidant and particulate load induced by inhalation of smoke. The studies also raised the likelihood that the lung’s response undergoes changes between 1 and 5 days of smoke exposure. Gene profiling is one approach to identifying pathways and processes that are changed by smoke in each genotype and sex. We therefore pursued studies of whole genome profiling to ask questions in an unbiased manner about the changes in gene expression induced by 1- and 5-day smoke exposure.

Our studies of gene expression tested the hypothesis that the pulmonary response to cigarette smoke, as measured by expression of exposure-response genes and the association scores of gene sets, is different between 1 and 5 days of cigarette smoke. We determined the changes in gene expression in order to evaluate in an unbiased manner how the lung adapts to acute cigarette smoke exposure. These changes in expression were then analyzed to identify the processes and mechanisms through which adaptation may be occurring, through gene set analysis. The most novel and exciting information comes from the opportunity to study two acute durations of smoke and to determine whether the lung’s response changes over this short interval.

Our study design compared mice exposed to cigarette smoke for a single session to mice exposed to five times that cumulative dose delivered over five consecutive days. Our study shows that after both 1 and 5 days of smoke exposure, the lung responds with the up- and downregulation of hundreds of genes. Interestingly, certain gene networks are upregulated after the first exposure to cigarette smoke but become downregulated by 5 days of exposure, such as processes regulating the ECM. In contrast, processes regulating the immune response are consistently downregulated after both 1 and 5 days of smoke. Furthermore, other gene sets representing the biological pathways of oxidative stress and xenobiotic responses are consistently upregulated in response to smoke. For example, at both 1 and 5 days there is a significant upregulation of NRF2-mediated cytoprotection to the oxidative stress response and of gene sets describing glutathione-mediated detoxification. Therefore, the gene expression profile and, specifically, the exposure response, is similar after 1 and 5 days of exposure, but contains important differences in gene expression representing biological functions that show the lung modulates its response to smoke.

To address concerns about replicability, we compared the lists of genotype-response genes at 1 and 5 days to previously reported genotype-response genes in βENaC and WT mice of the same age [35]. There was significant overlap between the published gene list and the 1 day (75% overlap) and 5 day (84% overlap) genotype-response gene lists (S1 Table). Therefore, the genotype results successfully replicate previous findings, validating both the biological results and the technical methods used to discover the response genes in this study. Additionally, these data support our observation that the genotype changes occur independently from the smoke exposure responses at these acute durations.

**Fig 6:**
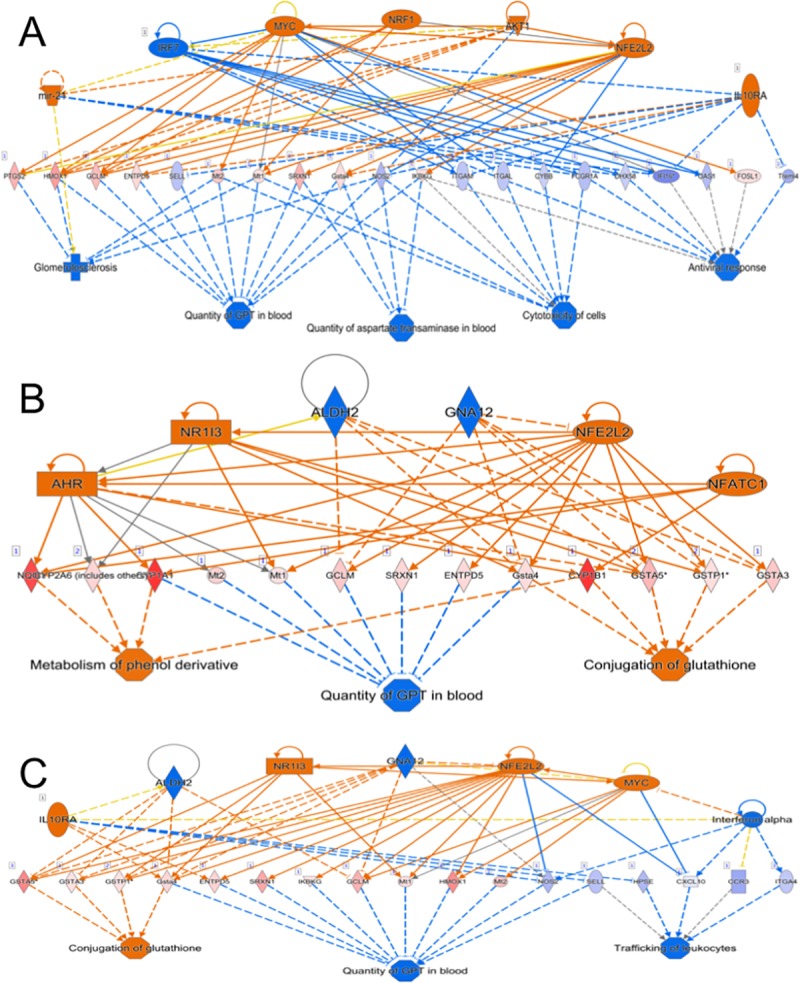
IPA network analysis investigating NRF2’s role in exposure-response genes after 1- and/or 5-day exposures. The red and blue coloration of the molecules in the middle row corresponds to the expression level: red indicates that the gene is upregulated due to smoke, and blue indicates downregulation due to smoke. (A): The regulator effect network map showing the NRF2-mediated oxidative stress response. This regulator was strongly predicted to regulate the pooled list of exposure-response genes significant after 1 and/or 5 days. The red and blue coloration reflects the expression level after 1 day of exposure. (B): The regulator effect network map showing the factors involved in regulating the cellular response, mediated by NFE2L2 (NRF2), after 1 day of exposure. (C): The regulator effect network map showing the factors involved in regulating the oxidative response, mediated by NFE2L2 (NRF2) after 5 days of exposure.

Certain biological functions respond to the presence of cigarette smoke after both 1- and 5-day exposures. In fact, most genes respond similarly between 1 and 5 days of exposure, as shown by the correlation coefficient of 0.79 in Fig 5A. Specifically, the xenobiotic and antioxidant responses are the most highly associated with exposure and are increased after both 1 and 5 days. These responses have previously been reported in the literature at chronic smoke durations for humans [52] and mice [28, 53], and observed even at sub-chronic (4 and 8 week) exposure durations in mice [54]. The gene sets that are upregulated in response to smoke after both 1 and 5 days involve diverse processes from oxidative stress and metabolic functions. These results provide insight into aspects of the lung’s response to smoke exposure that is likely independent of exposure duration. For example, the NRF2-mediated cytoprotective response to oxidative stress pathway is upregulated after 1 and 5 days of exposure (Fig 6A). Mice deficient in the gene that codes for Nrf2 (Nfe2l2) show accelerated and enhanced injury induced by cigarette smoke [55, 56]. After 1 day of smoke exposure, pathways involving cytoprotection of cells via Nrf2 are predicted to be changed (Fig 6B). By 5 days of exposure, the oxidative stress response pathways regulating glutathione conjugation via Nrf2 regulation are predicted (Fig 6C). Our data provide insight into which Nrf2-regulated genes may be mediating this protection. Alterations in antioxidant responses together with metabolic changes in the lung are well documented in response to cigarette smoke and have been implicated in the development of COPD [57]. Therefore, these responses are present and measurable after a single exposure to cigarette smoke and are maintained through consecutive repeated exposures, and they remain at chronic time points. Thus, Nrf2 is an important aspect of the mechanism through which smoke exposure regulates gene expression changes [55, 56].

Strikingly, the gene sets that were downregulated after both 1 and 5 days of exposure are involved in numerous aspects of immunity. These results are validated by the lack of an effect of cigarette smoke on the individual chemokines, cytokines and metalloproteases described in Tables 3 and 4. Changes in the immune system of the lung in response to chronic cigarette smoke exposure have previously been documented in humans [57], and have been found to be conserved in comparisons between human and mouse responses to chronic smoke [28, 58]. Decreases in the numbers of inflammatory cells and chemokines and immune suppression have also been well-documented as responses to smoke exposures [59–62].

The most interesting questions address differences that occur in response to a single dose of cigarette smoke to those resulting from consecutive repeated exposures. Exposure duration-dependent gene responses are denoted by a significant interaction between exposure and exposure duration. These genes are enriched in gene sets regulating biological processes such as the oxidative stress response through glutathione oxidation, metabolism pathways, xenobiotic responses, and the ECM biology (Table 8). ECM biology has long been associated with chronic smoke exposure [38, 47, 49, 63, 64]. However, a comparison of the response after acute smoke, and particularly after a single compared to five consecutive doses of cigarette smoke has not been previously assessed, to the best of our knowledge. Importantly, there are no genotype- or sex-associated pathways which act discordantly by exposure duration, suggesting that these changes are independent of exposure duration. We suggest that the different changes in response to exposure duration may represent the lungs’ attempt to adapt to repeated exposures, which ultimately results in the matrix changes and the effects of abnormal immune responses that is eventually manifested as chronic obstructive pulmonary disease.

Pathways and genes modulating the ECM showed a discordant response to exposure duration; although these pathways are initially upregulated in response to smoke after 1 day of exposure, they are downregulated by 5 days. This suggests that smoke rapidly induces genes, including structural genes coding for collagens and laminins, in response to acute cellular injury. By 5 days of smoke exposure, the cytoprotective effects of Nrf2 target activation and other antioxidant processes may result in less need for these ECM repair mechanisms at this point in the lung’s response.

ECM biology-related gene sets derived from Burgstaller and colleagues’ recent review of ECM biology [47] in the lung replicated the association with smoke and discordant behavior due to exposure duration: the ECM gene set containing all ECM components was significantly upregulated due to smoke after 1 day, but downregulated after 5 days (Table 10). ECM remodeling in response to chronic cigarette smoke has been recorded before in animal models [63, 65, 66]. Subsets of the ECM gene sets deal with different facets of ECM biology, including ECM components and their regulation and cell-matrix interactions, and provide information about the way the lung reshapes the ECM in response to cigarette smoke. The gene set composed of glycoprotein-encoding genes from the recent ECM review showed significant association with smoke and behaved discordantly with exposure duration: this gene set was significantly upregulated due to smoke after 1 day but downregulated after 5 days (Table 9). Furthermore, this association with smoke and the discordant changes by exposure duration was present in the glycoprotein subset only and not in the collagen or proteoglycan subsets, although the composite list of all components did respond similarly (Table 9). Additionally, there is evidence of an effect of estrogen in ECM remodeling in mice exposed to chronic cigarette smoke [64]. This corresponds with the enrichment of estrogen biosynthesis genes within the exposure-response gene list (Table 7).

The exposure-response genes identified after 1 and 5 days of smoke represent the lung’s response to a single and five consecutive daily doses of smoke. In order to explore how these responses compared to gene expression in the lung in an established environment of chronic smoke exposure, we compared these gene lists to DEGs identified in WT mice after six months of smoke exposure [51]. Miller and colleagues identified 111 genes that were differentially expressed in smoke- compared to sham-exposed WT mice. There was significant overlap between this list of DEGs and our results. After 1 day, 26 of the exposure-response genes identified in our study were also identified after 6 months of smoke exposure (p = 2e-24). After 5 days of smoke exposure, 40 of the exposure-response genes were also identified by Miller et al.’s study (p = 8.6e-45).

Nicotine is metabolized to cotinine and its breakdown products primarily in the liver, although the lung also contributes to cotinine production. Our data show that cotinine concentrations in the plasma were undetectable in all sham-exposed mice and increased after 1 and 5 days of smoke exposure. Curiously, the concentration of cotinine was actually less after 5 compared to 1 daily exposure. In humans, CYP2A6 is a critical enzyme in both nicotine and cotinine metabolism, and polymorphisms that affect its activity are critical in the rate of nicotine metabolism [67]. This particular cytochrome P450 gene is not expressed in mice. Rather, in C57Bl/6 mice, Cyp2a5 is the major nicotine metabolizing enzyme, responsible for 70–90% of the metabolism of nicotine to cotinine and cotinine to 3-hydroxycotinine [68–71]. Because transcriptional regulation is often conserved across tissues at the transcriptome level, we compared Cyp2a5 mRNA expression in the RNA isolated from lung tissue samples. Cyp2a5 mRNA expression is in fact increased 1.4-fold at both 1- and 5-day smoke exposures (q = 0.006 and 0.013, respectively). These studies provide a clear example of the impact of changes in gene expression on the host response to acute smoke exposure and how these changes very rapidly lead to diminution in the levels of a toxic mediator between 1 and 5 days.

Our studies of immune cell numbers and mediators showed differences between males and females in their response to cigarette smoke and to the thickened mucus found in βENaC mice (Tables 3 and 4, Figs 1 and 2). These studies, as well as observations made by others in studies of human disease [9, 11, 13, 14, 17, 19, 72–75], suggest that males and females respond differently to smoke. As personalized medicine becomes the standard of care, understanding the influence of sex on an individual’s unique experience of an established disease like COPD becomes increasingly important. We therefore hypothesized that there would be genotype- and sex-specific responses to acute smoke exposures. However, an interaction test to identify genes that responded differently to smoke depending on genotype or sex showed that these dependent relationships were not present in the dataset (S14 Table). This result could be a true negative result, or it could be that the sample size was too small to detect these interactions. While we did not find many significant dependent relationships between exposure and either genotype or sex, the expression of many exposure-response genes is further modulated by genotype and sex, independently. Additionally, these changes correlate almost perfectly between 1 and 5 days of smoke exposure (Fig 5), showing that there are no overall exposure length-dependent changes in the genotype- or sex-response genes. Taken together, the genotype- and sex-associated changes occur independently of smoke exposure and of exposure duration. Although individual genes may be responding in an exposure length-dependent manner, there is no evidence for exposure length-dependent sex or genotype differences in the gene profile changes at these acute exposure lengths.

This study looked at RNA from the total homogenized lung tissue, which is a very heterogeneous mixture of numerous cell types. These methods are ideal for discovery of smoke-response genes in the transcriptome that are highly expressed after acute smoke exposures and for generation of new hypotheses. However, this also creates the limitation that the methods did not allow for identification of the cell population of origin for each differentially expressed gene. Future studies could identify the source of these changes and the signaling pathways responsible for the functional changes.

Taken together, these studies demonstrate that within a short window of smoke exposure, significant inflammatory responses and changes in leukocyte kinetics occur within the pulmonary tissue and that these effects depend on the pre-existing health status of the tissue as well as the subject’s sex. Gene expression analysis comparing the lung’s response to the first and repeated consecutive cigarette smoke exposures provides information and generates hypotheses to help direct future research questions. Understanding the ways in which the lungs modulate their response to cigarette smoke after repeated exposures can contribute new information about the toxicology of smoke and eventually contribute to understanding the therapeutic potential in regulatory signaling pathways that are beneficial or detrimental to lung health.

## Supporting information

S1 Table Robustness of gene expression data confirmed using published gene lists for βENaC mice. Genotype-response genes after 1 and 5 days with a fold change greater than +/-1.3 that were identified in this study were compared with previously published DEGs in βENaC mice versus WT mice [35]. Orange coloring indicates that the gene was present in both our gene list(s) and the published study, while blue coloring indicates that this gene was not present on our genotype-response gene list(s).
(XLSX)

S2 Table Results for association of all genes and all variables after 1 day of exposure. This table includes the β coefficients, p values, and q values for association with exposure, genotype, and sex for every gene measured after 1 day of exposure.
(XLSX)

S3 Table Results for association of all genes and all variables after 5 days of exposure. This table includes the β coefficients, p values, and q values for association with exposure, genotype, and sex for every gene measured after 5 days of exposure.
(XLSX)

S4 Table Results for association of exposure-response genes with fold change>1.3 after 1 day of exposure. This table includes the subset of all significant exposure-response genes with a fold change greater than +/-1.3 after 1 day of exposure, with the corresponding β coefficients, p values, and q values for association with exposure, genotype, and sex.
(XLSX)

S5 Table Results for association of exposure-response genes with fold change>1.3 after 5 days of exposure. This table includes the subset of all significant exposure-response genes with a fold change greater than +/-1.3 after 5 days of exposure, with the corresponding β coefficients, p values, and q values for association with exposure, genotype, and sex.
(XLSX)

S6 Table Exposure-response genes with a significant duration-dependent response. This table includes the subset of all exposure-response genes significant at 1 and/or 5 days which were also significant (q<0.05) for a post-hoc interaction test between exposure and exposure duration. The table includes the B coefficient for exposure and the p value and q value of the interaction test for each gene with q<0.05. These genes were then put into GSEA’s overlap tool to assess for enrichment in any Canonical Pathways.
(XLSX)

S7 Table Summary of permutation testing results and significance thresholds for gene set analysis. This table summarizes the GSA score thresholds used to define significance (q<0.05).
(XLSX)

S8 Table GSA scores for exposure-associated gene sets after 1 day of exposure. All gene sets with significant association with exposure after 1 day are reported.
(XLSX)

S9 Table GSA scores for exposure-associated gene sets after 5 days of exposure. All gene sets with significant association with exposure after 5 days are reported.
(XLSX)

S10 Table GSA scores for genotype-associated gene sets after 1 day of exposure. All gene sets with significant association with genotype after 1 day of exposure are reported.
(XLSX)

S11 Table GSA scores for genotype-associated gene sets after 5 days of exposure. All gene sets with significant association with genotype after 5 days of exposure are reported.
(XLSX)

S12 Table GSA scores for sex-associated gene sets after 1 day of exposure. All gene sets with significant association with sex after 1 day of exposure are reported.
(XLSX)

S13 Table GSA scores for sex-associated gene sets after 5 days of exposure. All gene sets with significant association with sex after 5 days of exposure are reported.
(XLSX)

S14 Table Summary of linear model testing. This table summarizes the numbers of significant genes found for genotype and sex using the linear model. The last row shows that no genes had a significantly better fit (q<0.05) using an interactive model that shows exposure-dependent responses in genotype and sex when compared with an additive linear model describing only independent changes between exposure, genotype, and sex.
(XLSX)

S1 File Code for linear modeling and analysis. This file includes the R code for setting up and running the linear model, producing the heatmap of exposure-response genes, the post-hoc interaction test between exposure and exposure duration, the correlation plots for β coefficients at 1 and 5 days, and the code for determining the robustness of the genotype-response signature from published work.
(R) Click here for additional data file.

S2 File Code for gene set analysis. This file includes code for testing association between the Canonical Pathways gene sets and exposure, genotype, and sex after 1 and 5 days of exposure. This file also includes gene set testing for the custom gene sets involving ECM biology and association with exposure, genotype, and sex after 1 and 5 days of exposure.
(R) Click here for additional data file.

S3 File List of all Canonical Pathways gene sets. GMT file of all Canonical Pathways gene sets from GSEA used for gene set analysis (version 5.2).
(GMT)

S4 File List of custom ECM gene sets. GMT file of all custom ECM gene sets derived from published literature [47] used for gene set analysis.
(GMT)

S5 File List of custom βENaC gene sets. GMT file of all custom βENaC vs WT gene sets derived from published literature [35] and from our own linear model analysis at 1 and 5 days used for gene set analysis.
(GMT)
